# Genetic variants in genes involved in creatine biosynthesis in patients with severe obesity or anorexia nervosa

**DOI:** 10.3389/fgene.2023.1128133

**Published:** 2023-04-10

**Authors:** Luisa S. Rajcsanyi, Anne Hoffmann, Adhideb Ghosh, Birgit Matrisch-Dinkler, Yiran Zheng, Triinu Peters, Wenfei Sun, Hua Dong, Falko Noé, Christian Wolfrum, Beate Herpertz-Dahlmann, Jochen Seitz, Martina de Zwaan, Wolfgang Herzog, Stefan Ehrlich, Stephan Zipfel, Katrin Giel, Karin Egberts, Roland Burghardt, Manuel Föcker, Linus T. Tsai, Timo D. Müller, Matthias Blüher, Johannes Hebebrand, Raphael Hirtz, Anke Hinney

**Affiliations:** ^1^ Department of Child and Adolescent Psychiatry, Psychosomatics and Psychotherapy, University Hospital Essen, University of Duisburg-Essen, Essen, Germany; ^2^ Center for Translational Neuro- and Behavioral Sciences, University Hospital Essen, Essen, Germany; ^3^ Helmholtz Institute for Metabolic, Obesity and Vascular Research (HI-MAG) of the Helmholtz Zentrum München at the University of Leipzig and University Hospital Leipzig, Leipzig, Germany; ^4^ Institute of Food, Nutrition and Health, ETH Zurich, Schwerzenbach, Switzerland; ^5^ Department of Child and Adolescent Psychiatry and Psychotherapy, University Hospital of the RWTH Aachen, Aachen, Germany; ^6^ Department of Psychosomatic Medicine and Psychotherapy, Hannover Medical School, Hannover, Germany; ^7^ Department of Internal Medicine II, General Internal and Psychosomatic Medicine, University of Heidelberg, Heidelberg, Germany; ^8^ Translational Developmental Neuroscience Section, Division of Psychological and Social Medicine and Developmental Neurosciences, Faculty of Medicine, TU Dresden, Dresden, Germany; ^9^ Eating Disorders Research and Treatment Center, Department of Child and Adolescent Psychiatry, Faculty of Medicine, TU Dresden, Dresden, Germany; ^10^ Department of Psychosomatic Medicine and Psychotherapy, Medical University Hospital Tübingen, Tübingen, Germany; ^11^ Center of Excellence in Eating Disorders KOMET, Tübingen, Germany; ^12^ Department of Child and Adolescent Psychiatry, Psychosomatics and Psychotherapy, University Würzburg, Würzburg, Germany; ^13^ Child and Adolescent Psychiatry Clinic, Oberberg Fachklinik Fasanenkiez Berlin, Berlin, Germany; ^14^ Department of Child and Adolescent Psychiatry, University Hospital Münster, Münster, Germany; ^15^ Division of Endocrinology, Diabetes and Metabolism, Department of Medicine, Beth Israel Deaconess Medical Center, Harvard Medical School, Boston, MA, United States; ^16^ Institute for Diabetes and Obesity, Helmholtz Diabetes Center, Helmholtz Zentrum München, München, Germany; ^17^ German Center for Diabetes Research (DZD), Neuherberg, Germany; ^18^ Department of Pediatrics, Division of Rare Diseases, and CeSER, Ruhr-University Bochum, Bochum, Germany; ^19^ Department of Pediatric Endocrinology and Diabetology, Clinic for Pediatrics II, University Hospital Essen, Essen, Germany

**Keywords:** GWAS, creatine metabolism, *in silico*, BAT, TDT

## Abstract

Increased thermogenesis in brown adipose tissue might have an obesity-reducing effect in humans. In transgenic mice, depletion of genes involved in creatine metabolism results in disrupted thermogenic capacity and altered effects of high-fat feeding on body weight. Data analyses of a sex-stratified genome-wide association study (GWAS) for body mass index (BMI) within the genomic regions of genes of this pathway (*CKB*, *CKMT1B*, and *GATM*) revealed one sex-dimorphic BMI-associated SNP in *CKB* (rs1136165). The effect size was larger in females than in males. A mutation screen of the coding regions of these three candidate genes in a screening group (192 children and adolescents with severe obesity, 192 female patients with anorexia nervosa, and 192 healthy-lean controls) identified five variants in each, *CKB* and *GATM*, and nine variants in the coding sequence of *CKMT1B*. Non-synonymous variants identified in *CKB* and *CKMT1B* were genotyped in an independent confirmation study group (781 families with severe obesity (trios), 320 children and adolescents with severe obesity, and 253 healthy-lean controls). *In silico* tools predicted mainly benign yet protein-destabilizing potentials. A transmission disequilibrium test in trios with severe obesity indicated an obesity-protective effect of the infrequent allele at rs149544188 located in *CKMT1B*. Subsequent correlation analyses in 1,479 individuals of the Leipzig Obesity BioBank revealed distinct correlations of *CKB* with the other two genes in omental visceral adipose tissue (VAT) and abdominal subcutaneous adipose tissue (SAT). Furthermore, between-subject comparisons of gene expression levels showed generally higher expressions of all three genes of interest in VAT than in SAT. Future *in vitro* analyses are needed to assess the functional implications of these findings.

## 1 Introduction

Globally, the prevalence of obesity is rising continuously (Williams et al., 2015; Schienkiewitz et al., 2017; Janssen et al., 2020; Hinney et al., 2022). By 2030, approximately 50% of the global population is projected to exhibit a body mass index (BMI) greater than 30 kg/m^2^ and will thus be considered obese (World Health Organisation, 2000; Finkelstein et al., 2012; González-Muniesa et al., 2017; Ward et al., 2019). The global prevalence of severe obesity in children is approximately 2%–6% (Hinney et al., 2022).

A proposed mechanism to counteract obesity is to increase energy expenditure *via* enhanced activity of the brown adipose tissue (BAT), commonly referred to as adaptive thermogenesis (Chouchani et al., 2019; Pan et al., 2020). In addition to the known uncoupling protein 1 (UCP1)-dependent pathway, an alternative creatine-dependent mechanism has been shown to affect thermogenesis (Chouchani et al., 2019). A reduction in creatine levels through the inhibition of its biosynthesis diminished thermogenesis and energy expenditure, leading to a weight gain (Kazak et al., 2015; Kazak et al., 2017). Upon high-fat feeding, mice in which the first and thus rate-restricting enzyme of the creatine biosynthesis, glycine amidinotransferase (GATM), was fat-selectively deprived, showed an increased body weight (Kazak et al., 2017). GATM transports a guanido group from arginine to glycine resulting in guanidinoacetic acid. The latter is a direct creatine precursor (Morris, 2004).

Throughout creatine biosynthesis, creatine kinases catalyze the reversible transfer of a phosphate from adenosine triphosphate (ATP) to creatine, resulting in phosphocreatine (PCr; Hettling and van Beek, 2011). Mice in which either creatine kinase B (Ckb) or creatine kinase mitochondrial 1 (Ckmt1) was knocked out (KO) showed a clinically mild phenotype. These mice were fertile but had behavioral abnormalities, such as reduced spatial learning abilities (Jost et al., 2002; Streijger et al., 2004). It has been suspected that the respective other kinase compensates for the KO, and thus, a double-KO mouse model was studied (Streijger et al., 2005). In these double-KO mice, lacking both creatine kinases (CK−/−), an 18%–20% lower body weight as well as a 7% reduction of BAT was found (Streijger et al., 2005; Streijger et al., 2009). In addition, these mice exhibited problems maintaining their body temperature, which was proposed to be due to a communication loss of thermogenesis-associated pathways in the hypothalamus induced by reduced levels of creatine kinases (Streijger et al., 2009). However, brown adipocyte-selective inhibition of Ckb in mice predisposes to obesity due to a reduction in thermogenic capacity (Rahbani et al., 2021). Similarly, upon depletion of the creatine transporter (CrT) in fat depots, mice gained more weight than their wild-type littermates when fed a high-fat diet. This CrT KO led to reduced creatine and phosphocreatine levels in adipocytes and diminished energy expenditure. Accordingly, in purified human subcutaneous adipocytes, the expression of CrT correlated with a decreased BMI (Kazak et al., 2019).

Previous studies have reported differences in the regulation of BAT activity and body weight between males and females (e.g., Shi and Clegg, 2009; Kaikaew et al., 2021). Animal models have demonstrated that sex hormones might mediate the sexually dimorphic regulation. Accordingly, androgens inhibited BAT activity, whereas estrogens showed a stimulatory effect. Generally, female rodents were found to have a higher prevalence of active BAT than males (Kaikaew et al., 2021). In human BAT, conclusive evidence is still lacking. Several studies reported that BAT activity and mass are higher in females than males, while others were unable to detect any differences between the sexes (Kaikaew et al., 2021).

In contrast, sex differences at the genetic level of body weight regulation have been detected in a genome-wide association study (GWAS) for waist-to-hip ratio adjusted for BMI (Pulit et al., 2019). Higher heritability and larger effects of genetic variants were found in women compared to men, while approximately 1/3 of all GWAS signals generally displayed a sexually dimorphic effect (Pulit et al., 2019). Additionally, several studies have provided evidence for genetic overlaps between BMI and anorexia nervosa (AN; Bulik-Sullivan et al., 2015; Hinney et al., 2017; Watson et al., 2019; Zheng et al., 2022). AN is a severe psychiatric disorder marked by an extremely reduced body weight and a distorted body image (Hübel et al., 2019; Mitchell and Peterson, 2020). It mainly occurs in females and exhibits one of the highest mortality rates in psychiatric disorders (e.g., Arcelus et al., 2011). As alleles predisposing to AN are also associated with a lower BMI (Hinney et al., 2017), genetic analyses in both extremes of the BMI range may help to improve the general understanding of body weight regulation.

Consequently, we aimed to analyze sex-stratified effects of single nucleotide polymorphisms (SNPs) previously identified in GWAS for BMI (Pulit et al., 2019) or AN (Watson et al., 2019) and located in the genomic regions of *CKB*, *CKMT1B*, and *GATM*. Subsequently, we sequenced the coding regions of these genes in children and adolescents with severe obesity and healthy-lean controls. Given the results of our sex-stratified analysis, the coding sequence (CDS) of *CKB* was further screened in female patients with AN. Non-synonymous variants identified in *CKB* and *CKMT1B* were genotyped in additional independent samples. *In silico* analyses were conducted to explore putative functional implications. Next, we conducted correlation analyses of the genes of interest with several metabolic and anthropometric parameters, such as BMI, body weight, height and serum leptin concentrations, as well as between-subject comparisons based on gene expression levels, in omental visceral adipose tissue (VAT) and abdominal subcutaneous adipose tissue (SAT) of 1,479 individuals of the Leipzig Obesity BioBank (LOBB).

## 2 Materials and methods

### 2.1 GWAS-related sex-specific analyses

To evaluate sex-specific effects of genetic variants within the genes of interest (*CKB*, *CKMT1B,* and *GATM*), data from a sex-stratified meta-analysis of GWAS for BMI (Pulit et al., 2019) were utilized (https://zenodo.org/record/1251813#.YbsPznyZNaR; downloaded on 8 June 2022). A total of 434,794 female and 374,756 male samples as well as 27, 381, 302 SNPs were included (Pulit et al., 2019). Information regarding the genomic position, rsID, minor allele frequency (MAF), standard error (SE), beta and *p*-values (p) of the SNPs located within the genes and a 500 kb region adjacent to the genes were extracted from the datasets. Given the spatial proximity of *GATM* and *CKMT1B*, these genes were analyzed collectively within one region (see Table 1). After assessing whether the effect alleles for males and females were homogeneously assigned, palindromic SNPs with a MAF between 0.48 and 0.52 were excluded. The SNPs’ effect directions were subsequently harmonized to eliminate strand issues by inverting the beta values of SNPs tested for their major allele. Next, z-scores were calculated for each SNP (see Eq. 1; Khramtsova et al., 2019; Zheng et al., 2022). Furthermore, additional data from a GWAS for AN (Watson et al., 2019) were obtained (https://www.med.unc.edu/pgc/download-results/; downloaded on 21 October 2021). However, as no information on MAF was available in this dataset, effect size harmonization was omitted. SNPs were considered trait-associated if they exhibited a GWAS-derived *p* < 5*10^–8^.

$$z-score=\frac{{beta}_{female}-{beta}_{male}}{\sqrt{{SE^{2}}_{female}+{SE^{2}}_{male}}}$$ (1)

**TABLE 1:** Genomic positions investigated in the sex-stratified analyses. Since *CKMT1B* and *GATM* are located in close proximity, these two genes were investigated collectively within one region (*). The analyzed regions refer to GRCh37, as all GWAS data were exclusively available for this genome version.

Gene	Chr	Position	±500 kb region	Investigated region
*CKB*	14	103,986,004–103,989,167	103,486,004–104,489,167	103,486,004–104,489,167
CKMT1B*	15	43,885,055–43,891,604	43,385,055–44,391,604	43,385,055–46,194,425*
GATM*	15	45,653,322–45,694,425	45,153,322–46,194,425	43,385,055–46,194,425*

SNPs were defined to have a sexually dimorphic effect if they exhibited an absolute z-score of at least 3 (|z-score| ≥ 3), as described previously (Khramtsova et al., 2019).

### 2.2 Screening and confirmation study group for the mutation screen

We sequenced the DNA of a screening group consisting of 192 children and adolescents with severe obesity (age: 13.88 ± 2.50 years; BMI: 32.36 ± 6.04 kg/m^2^; all had a BMI percentile >90th; 182 had a BMI >97th percentile; see Table 2; Hinney et al., 2007; Scherag et al., 2010), 192 patients with anorexia nervosa (acute or recovered; age: 21.85 ± 10.60 years; BMI: 16.38 ± 3.12 kg/m^2^; see Table 2; Boraska et al., 2012; Watson et al., 2019) and 192 healthy-lean controls (age: 25.55 ± 3.91 years; BMI: 18.38 ± 1.10 kg/m^2^; see Table 2; Hinney et al., 2007). Additional genotyping was performed in a confirmation study group of 781 families comprising a child or adolescent with severe obesity and both biological parents (referred to as trios; age of offspring: 13.45 ± 3.11 years; BMI of offspring: 32.15 ± 5.92 kg/m^2^; see Table 2) from a family-based obesity GWAS, 320 children and adolescents with severe obesity (age: 14.60 ± 4.24 years; BMI: 33.77 ± 7.00 kg/m^2^, see Table 2) and 253 healthy-lean controls (age: 26.40 ± 6.84 years; BMI: 17.85 ± 1.11 kg/m^2^; see Table 2) from a case-control GWAS for obesity (Hinney et al., 2007; Scherag et al., 2010). The 781 trios were composed of a child or adolescent with severe obesity (BMI percentile 90th) and both biological parents. Over 90% of the offspring (723; 92.57%) had a BMI at or above the 97th BMI percentile. The mean BMI of the 1,562 parents was 30.40 kg/m^2^ (standard deviation: 6.3 kg/m^2^). Of these, 724 (46.35%) had a BMI above 30 kg/m^2^, while 822 (52.62%) showed a BMI lower or equal 30 kg/m^2^. For 16 parents, no BMI data was available. Written informed consent was given by all participants of the screening and confirmation study groups and, in the case of minors, by their parents. This study was further approved by the Ethics committees of the respective universities and institutions and was performed in accordance with the *Declaration of Helsinki*.

**TABLE 2:** Phenotypic data of the screening and confirmation study groups. The initial screening samples subjected to the mutation screen consisted of 192 children and adolescents with severe obesity or AN (acute or recovered) and 192 healthy-lean controls (Hinney et al., 2007; Scherag et al., 2010; Boraska et al., 2012; Watson et al., 2019). Additional genotyping was performed in an independent confirmation study group based on family-based samples consisting of the child or adolescent with severe obesity and both biological parents (trios) as well as additional individuals with severe obesity or normal weight previously recruited for a case-control GWAS for obesity (Hinney et al., 2007; Scherag et al., 2010). Phenotypic data regarding BMI and age were missing for 16 parents. n: number of samples. SD: standard deviation.

Sample	Status		N	% Female	Age in years: Mean (SD)	BMI in kg/m^2^: Mean (SD)	BMI-SDS_LMS_: Mean (SD)
Screening samples	Children and adolescents with severe obesity		192	56	13.88 (2.50)	32.36 (6.04)	2.60 (0.54)
	Patients with AN	total	192	100	21.85 (10.60)	16.38 (3.12)	−2.61 (1.86)
		acute	141	100	18.44 (8.70)	14.90 (1.85)	−3.20 (1.79)
		recovered	51	100	31.28 (9.68)	20.47 (2.09)	−0.98 (0.79)
	Controls		192	45	25.55 (3.91)	18.38 (1.10)	−2.21 (0.46)
Confirmation samples	Family-based samples	Children and adolescents with obesity	781	55	13.45 (3.11)	32.15 (5.92)	2.65 (0.53)
		Parents	1,562	50	42.60 (6.00)	30.38 (6.29)	0.77 (1.12)
	Samples from an obesity GWAS	Children and adolescents with obesity	320	58	14.60 (4.24)	33.77 (7.00)	2.69 (0.56)
		Controls	253	74	26.40 (6.84)	17.85 (1.11)	−2.32 (0.66)

Furthermore, the European, non-Finnish study group extracted from the Genome Aggregation Database (gnomAD; v2.1.1; GRCh37; Karczewski et al., 2020; https://gnomad.broadinstitute.org/; see Supplementary Table S3) was used as an additional control population. Data from either sex were considered as controls for the study group with severe obesity, while data from females served as controls for the screening group of exclusively female patients with AN. Phenotypic data regarding the gnomAD controls and genotype data pertaining to specific variants were unavailable. Additionally, the number of samples varied for each variant.

### 2.3 Study group for gene expression and correlation analyses

For gene expression and correlation analyses (based on RNA-sequencing data), paired omental visceral and abdominal subcutaneous adipose tissues were collected from 1,479 individuals of the Leipzig Obesity BioBank (LOBB). Individuals were either non-obese (n = 31; 51.6% female; age: 55.8 ± 13.4 years old; BMI: 25.7 ± 2.7 kg/m^2^) or had obesity (n = 1,448; 71.2% female; age: 46.9 ± 11.7 years old; BMI: 49.2 ± 8.3 kg/m^2^). Tissue samples were collected during elective laparoscopic abdominal surgery as described before (Langhardt et al., 2018). Laboratory measurements of body composition and metabolic parameters were obtained as detailed previously (Kloting et al., 2010). The study was approved by the Ethics Committee of the University of Leipzig (approval no: 159-12–21052012) and performed in accordance with the *Declaration of Helsinki*. All individuals gave written informed consent before participating in the study.

### 2.4 Mutation screen

The coding region of the genes *CKB* (chr14: 103,519,667–103,552,833; GRCh38; ENST00000348956.7), *CKMT1B* (chr15: 43,593,054–43,604,901; GRCh38; ENST00000441322.6), and *GATM* (chr15: 45,361,124–45,402,327; GRCh38; ENST00000396659.8) were Sanger sequenced in 192 children and adolescents with severe obesity and 192 healthy-lean controls (see Table 2). The CDS of *CKB* was further analyzed in 192 female patients with AN (see Table 2). The respective genomic fragments were amplified by performing polymerase chain reactions (PCR; Veriti96-well Thermal Cycler, Applied Biosystems, Foster City, CA, United States) with coding region-specific primers (see Supplementary Table S1). For the amplification of the CDS of *CKMT1B*, a nested PCR approach was conducted (see Supplementary Table S1). Confirmation of the desired fragments was achieved by 2.5% agarose gel electrophoresis. Subsequent Sanger sequencing was performed by MicroSynth Seqlab GmbH in Göttingen, Germany. Sequence analysis and genotype assignment were conducted independently by at least two experienced scientists using the SeqMan Pro software (version: 11.0.0; DNAstar, Inc., Madison, WI, United States). Discrepancies were solved by either reaching consensus or by re-sequencing.

### 2.5 Genotyping in a larger confirmation study group

**FIGURE 1:**
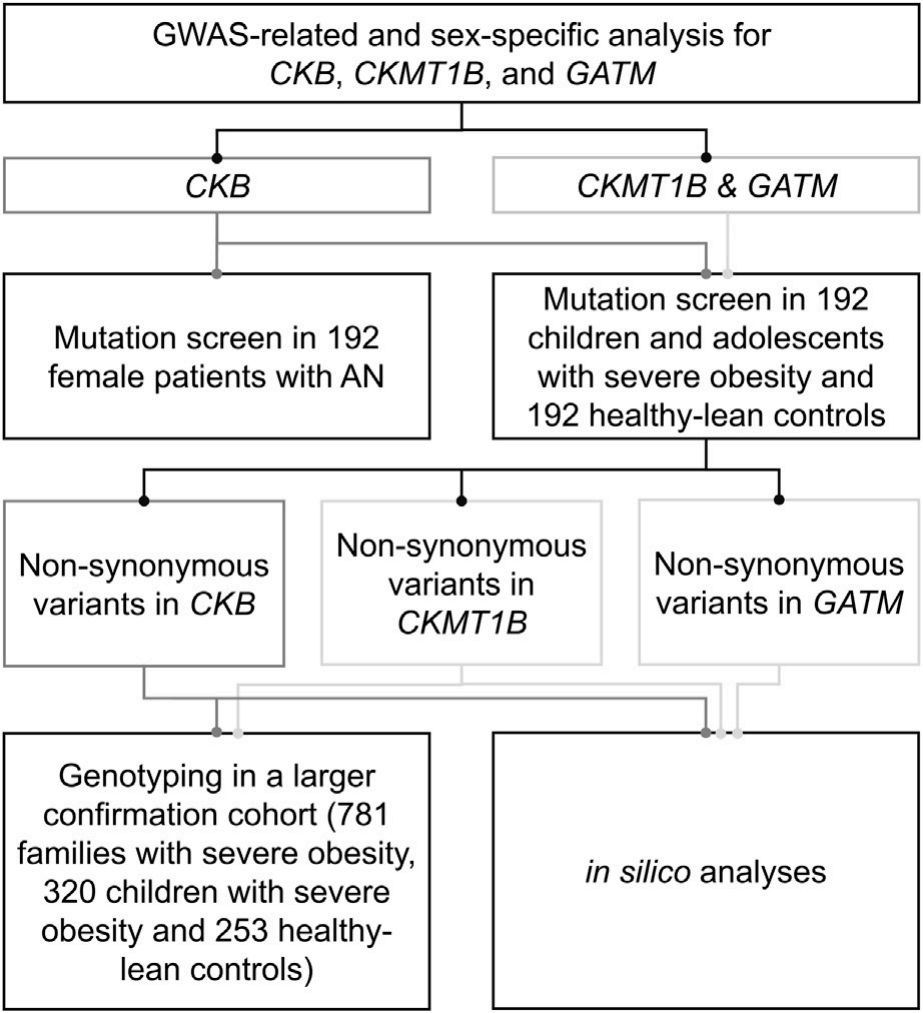
Experimental procedure of the genetic study. The genetic study comprised sex-specific analyses for *CKB*, *CKMT1B* and *GATM* as well as a mutation screen in children and adolescents with severe obesity, healthy-lean controls, and patients with anorexia nervosa. *In silico* analyses were used to predict the functional implications of the detected variants.

The *CKB*- and *CKMT1B*-located non-synonymous variants identified in patients with severe obesity were additionally genotyped in a larger independent confirmation study group, either by a TaqMan assay (ThermoFisher Scientific, Inc., Waltham, MA, United States) or a restricted fragment length polymorphism (RFLP) analysis (see Figure 1). Therefore, 781 trios with severe obesity as well as 320 children and adolescents with severe obesity and 253 healthy-lean controls from a previous GWAS (Hinney et al., 2007; Scherag et al., 2010) were analyzed (see Table 2). Custom TaqMan assays were designed for the variants rs758572075 (assay ID: ANXGZUF) and rs1230355611 (assay ID: ANYMVED). For the SNP rs146047573, a pre-designed assay was ordered (assay ID: C_162127713_10). All TaqMan assays were performed on the StepOnePlus Real-Time PCR system by Applied Biosystems (Foster City, CA, United States). Genotyping of SNP rs149544188 was performed by subjecting the samples to the restriction enzyme BsaHI (R0556S; New England Biolabs GmbH; Ipswich, MA, United States). Genotypes were assigned by at least two experienced scientists, while discrepancies were solved by either reaching consensus or by repeating the experiments.

### 2.6 Statistics

To exclude putative genotyping errors, the Hardy-Weinberg-Equilibrium (HWE) was checked. For two variants, a significant deviation of the HWE was detected. Consequently, the respective genotyping was re-checked and repeated. Once genotyping errors were excluded, the corresponding variants remained in the analyses. Furthermore, the transmission disequilibrium test (TDT) was conducted in the 781 trios with severe obesity for one variant analyzed in the confirmation study group (see Table 2; Spielman et al., 1993). Next, putative disease associations with either obesity or AN were assessed by a two-sided chi-square test or a two-sided Fisher’s exact test using IBM SPSS Statistics software (version 28.0.0.0). Associations were considered nominally associated if *p* < 0.05.

### 2.7 *In silico* analyses

All identified variants were subsequently analyzed by various *in silico* tools regarding their pathogenic implications, conservation (MutationTaster2021; Steinhaus et al., 2021), and potential to alter splice sites (ESEfinder, Cartegni et al., 2003; SpliceMan; Lim and Fairbrother, 2012). The impact of non-synonymous variants on the protein structure was examined with the tool PANTHER-PSEP (position-specific evolutionary preservation; Tang and Thomas, 2016) and the Cologne University Protein Stability Analysis Tool (CUPSAT, Parthiban et al., 2006). The following protein data bank (PDB) reference protein structures were utilized: CKB - 3DRE (Bong et al., 2008), CKMT1B- 1QK1 (Eder et al., 2000), and GATM - 2JDW (Humm et al., 1997).

### 2.8 Gene expression and correlation analyses

RNA-sequencing (RNA-seq; rRNA-depleted, single-end) data from the LOBB cohort were generated based on a SMARTseq protocol (Picelli et al., 2014). In brief, RNA enrichment and reverse transcription were conducted with oligo (dT) and TSO primers. cDNA amplification was carried out with ISPCR primers, and cDNA was processed with Tn5 using the Nextera DNA Flex kit. RNA libraries were sequenced on a Novaseq 6,000 instrument at the Functional Genomics Center Zurich (FGCZ), Switzerland.

The raw reads were adapter trimmed and checked for sufficient quality by applying Fastp (v0.20.0; Chen et al., 2018), permitting a minimum read length of 18 nts and a quality cut-off of 20. Aligned to the human reference genome (GRCh38.p13), gene level expression quantification (gene model definition from GENCODE release 32) was carried out using Kallisto (v0.46; Bray et al., 2016). Samples with more than 20 million read counts were downsampled to 20 million read counts using the R package ezRun (v3.14.1; https://github.com/uzh/ezRun). Data were homoscedastically normalized with respect to library size using the variance-stabilizing transformation implemented by DESeq2 (v1.32.0; Love et al., 2014). To effectively neutralize the effects of *in vitro* RNA degradation, normalized counts were calibrated with transcript integrity numbers (TINs; Wang et al., 2016). TINs were estimated using the R package RSeQC (v4.0.0; Wang et al., 2012). Finally, the normalized data were adjusted for the gender and age batches.

Correlation analyses were performed by determining Pearson’s correlation coefficient and a confidence interval of 0.95. Between-subject comparisons for the genes were conducted with a non-parametric one-way Kruskal–Wallis ANOVA and pairwise Dunn’s test utilizing the R package ggstatsplot (v0.9.1; Patil, 2021). *p*-values were corrected for multiple inference using the Hommel’s method (Hommel, 1988). Adjusted two-sided *p*-values (p_adj._) < 0.05 were considered significant. Analyses were performed under R version 4.2.1.

## 3 Results

### 3.1 One *CKB*-located SNP revealed a sex-dimorphic effect on BMI

**FIGURE 2:**
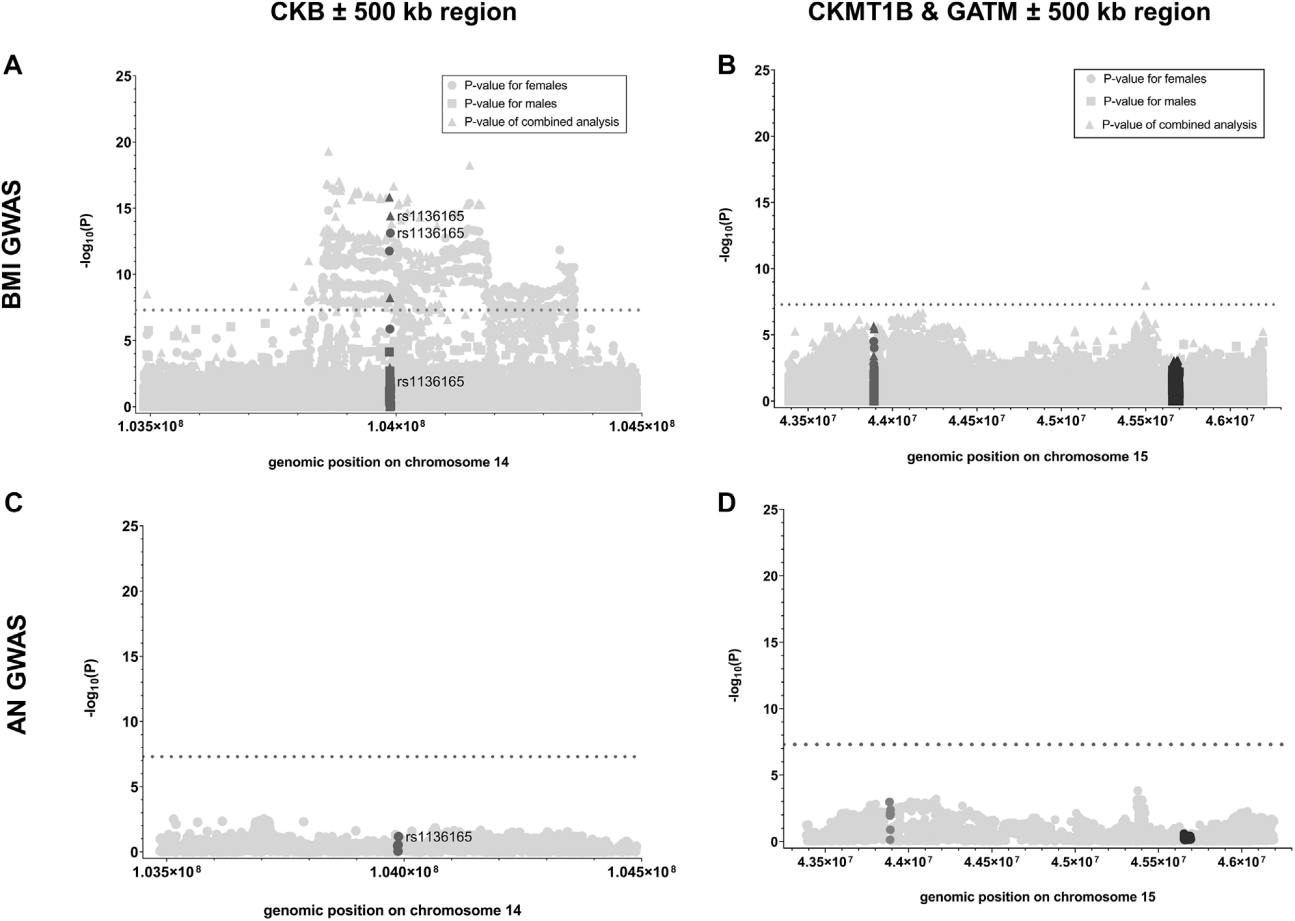
*p*-values of SNPs analyzed in GWAS for BMI or AN located within the genes of interest and their adjacent regions. Here, the negative logarithmic *p*-values extracted from the sex-stratified BMI GWAS [Pulit et al., 2019; **(A, B)**] and the GWAS for AN (Watson et al., 2019; **(C, D)** are plotted against the genomic positions of the SNPs located within *CKB* **(A, C)**; dark gray), *CKMT1B* [**(B, D)**; dark gray] and *GATM* [**(B, D)**; black] as well as their 500 kb adjacent regions (light gray; GRCh37; see Table 1; Supplementary Table S2). For the data regarding the sex-stratified BMI GWAS, the negative logarithmic *p*-values are presented for the female (dot), male (square), and combined (triangle) analyses. The threshold for genome-wide significance is indicated by the dotted lines (-log_10_(p) > 7.3).

**FIGURE 3:**
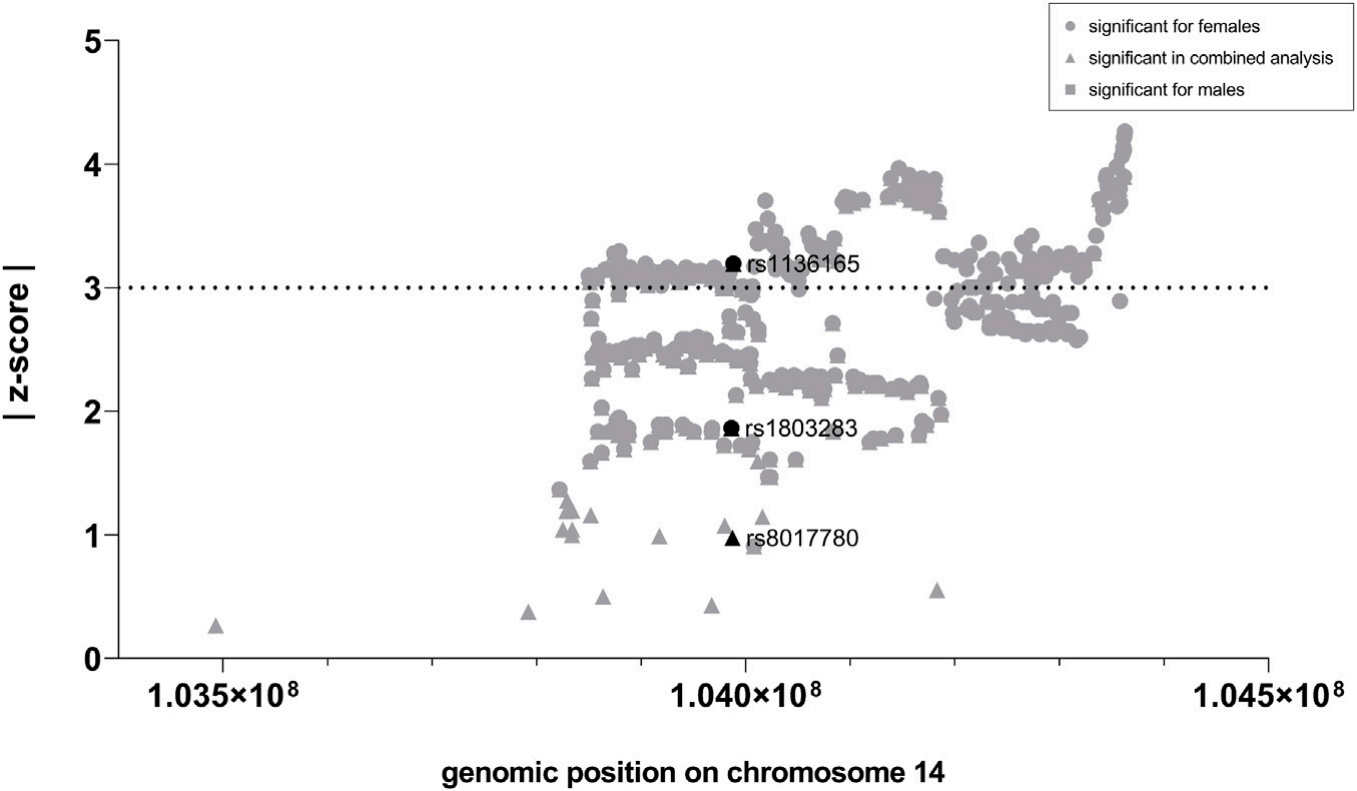
Z-scores for BMI-associated SNPs located in *CKB* and the 500 kb adjacent region. Here, the absolute z-scores were plotted against the chromosomal position of each SNP (GRCh37). After the extraction of the sex-stratified BMI GWAS data (Pulit et al., 2019), ambiguous SNPs were removed. Subsequently, the effect directions were harmonized, and the z-scores were calculated for each genome-wide and thus BMI-associated SNP within the region of *CKB* (black) and its 500 kb surrounding region (gray). A SNP is considered sex-dimorphic if |z-score| ≥ 3.

First, we analyzed whether GWAS-identified SNPs for BMI in *CKB*, *CKMT1B*, and *GATM* show sexually dimorphic effects. In the investigated GWAS (Pulit et al., 2019), a total of 24 SNPs were located in the genomic region of *CKB*, while 35 and 256 were encompassed in the regions of *CKMT1B* and *GATM*, respectively (see Supplementary Table S2). Notably, only *CKB* contained three SNPs (rs1803283, rs8017780, and rs1136165) that were associated with BMI (GWAS-derived *p* < 5*10^–8^) when both sexes were analyzed concomitantly. Two of these variants (rs1803283 and rs1136165) showed a significant BMI association in females (see Figure 2A), with their minor alleles predisposing to a lower BMI. No BMI-associated SNPs were detected in the region of *CKMT1B* and *GATM* (see Figure 2B; Supplementary Table S2). Calculating the z-scores with harmonized data of BMI-associated SNPs in *CKB* revealed that one variant (rs1136165; 4.17% of all *CKB*-located SNPs included in the GWAS) showed a more than 3-fold standard deviation aberration of effect sizes between females and males (absolute z-score = |3.196|; *p* = 0.0014; see Figure 3; Supplementary Table S2). As the tested allele at rs1136165 was solely associated with BMI in females (GWAS-derived *p* = 7.51*10^–14^ for tested allele T; GWAS-derived beta = 0.02 for tested allele T; Pulit et al., 2019) and not in males (GWAS-derived *p* = 0.013 for tested allele T; GWAS-derived beta = 0.008 for tested allele T; Pulit et al., 2019), this sexual dimorphism indicates a stronger BMI-altering effect in females than males. In the 500 kb region surrounding *CKB*, 225 SNPs (2.05% of all SNPs in this ±500 kb region) showed a significant sex-dimorphic effect (z-score > |3|). The highest z-score in this adjacent region was detected for rs72712857 (z-score = |4.27|; *p* = 0.00002; see Supplementary Table S2), which is exclusively BMI-associated in females (GWAS derived *p* = 1.44*10^–9^) (Pulit et al., 2019).

Given the lack of BMI associations within *CKMT1B* and *GATM* and adjacent regions (±500 kb), we solely calculated the z-score for one SNP that was associated with BMI in the sex combined analysis (rs765787; GWAS-derived *p* = 1.80*10^–9^) located in the 500 kb region surrounding the two genes of interest (*CKMT1B* and *GATM*; see Figure 2B; Supplementary Table S2). However, no sex-specific effect was detected (z-score = |0.38|, *p* = 0.71). Likewise, in the GWAS data for AN (Watson et al., 2019), we did not find SNPs associated with AN within the gene regions of *CKB*, *CKMT1B,* and *GATM* or in adjacent areas (see Figures 2C,D; Supplementary Table S2). Thus, we did not further analyze putative sex-specific effects within this dataset.

### 3.2 Mutation screen of *CKB*

Our sex-stratified analyses suggested a sex-dimorphic effect of the variant rs1136165 (p.Arg152=) located within *CKB* (see Figures 2, 3; Supplementary Table S2). Considering this GWAS hit and the observation that 1) AN and BMI share certain loci (Bulik-Sullivan et al., 2015; Hinney et al., 2017; Zheng et al., 2022), 2) AN mainly affects women (Hübel et al., 2019; Mitchell and Peterson, 2020), and 3) our GWAS-related sex-specific analyses may have been affected by insufficient power due to the limited sample size of the AN GWAS, we performed a mutation screen of *CKB*’s CDS in 192 female patients with AN (acute or recovered) as well as in 192 children and adolescents with severe obesity and 192 healthy-lean controls (see Table 2).

In total, we observed five variants (rs762206402, rs1136165, rs200890896, rs146047573, and rs1803283) in the CDS of *CKB*, one of which is non-synonymous (rs146047573, p.Tyr269Cys; see Table 3). All variants fulfilled the HWE.

**TABLE 3:** Identified genetic variants in the investigated study groups. The results include the screening groups of 192 children and adolescents with severe obesity as well as 192 patients with AN and 192 healthy-lean controls. The sample sizes may vary if the variant was genotyped in an additional confirmation group consisting of 781 trios with severe obesity, 320 independent children and adolescents with severe obesity, and 253 healthy-lean control samples (see Table 2). Non-synonymous variants are shown in bold. The represented minor allele is based on the forward strand (*). 11: Homozygous wild type; 12: heterozygous, 22: homozygous alternative allele. MAF: minor allele frequency.

Gene	Variant	Minor allele*	Amino acid exchange	Study group	Patients with obesity				Patients with AN				Healthy-lean controls			
					11	12	22	MAF	11	12	22	MAF	11	12	22	MAF
*CKB*	rs762206402	C	Pro112=	Screening	191	1	0	0.003	192	0	0	0	192	0	0	0
	rs1136165	G	Arg152=	Screening	31	88	73	0.39	28	88	76	0.38	29	96	67	0.40
	rs200890896	G	Ala175=	Screening	192	0	0	0	191	1	0	0.003	192	0	0	0
	**rs146047573**	C	**Tyr269Cys**	Screening	191	1	0	0.003	192	0	0	0	192	0	0	0
				Confirmation	1,100	1	0	0.0005	-	-	-	-	253	0	0	0
				Both	1,291	2	0	0.0008	-	-	-	-	445	0	0	0
	rs1803283	C	Glu364=	Screening	30	84	78	0.38	25	91	76	0.37	28	92	72	0.39
*CKMT1B*	g.43595061C/T	T	Thr79=	Screening	190	2	0	0.005	-	-	-	-	190	2	0	0.005
	g.43595385C/T	T	Asp120=	Screening	191	1	0	0.003	-	-	-	-	192	0	0	0
	g.43595451G/A	A	Thr142=	Screening	191	1	0	0.003	-	-	-	-	192	0	0	0
	**rs1230355611**	T	**Arg184Stop**	Screening	192	0	0	0	-	-	-	-	191	1	0	0.003
				Confirmation	1,100	1	0	0.0005	-	-	-	-	253	0	0	0
				Both	1,292	1	0	0.0004	-	-	-	-	444	1	0	0.001
	**rs758572075**	C	**Val191Ala**	Screening	191	1	0	0.003	-	-	-	-	190	2	0	0.005
				Confirmation	1,100	1	0	0.0005	-	-	-	-	253	0	0	0
				Both	1,291	2	0	0.0008	-	-	-	-	443	2	0	0.002
	rs9571	G	Lys352=	Screening	188	4	0	0.01	-	-	-	-	188	4	0	0.01
	rs13234	C	Ala362=	Screening	187	5	0	0.013	-	-	-	-	184	7	1	0.02
	**rs149544188**	A	**Arg399His**	Screening	190	2	0	0.005	-	-	-	-	191	1	0	0.003
				Confirmation	1,090	11	0	0.005	-	-	-	-	250	3	0	0.006
				Both	1,280	13	0	0.005	-	-	-	-	441	4	0	0.004
	rs144820945	G	Gly403=	Screening	188	4	0	0.01	-	-	-	-	188	4	0	0.01
*GATM*	**rs1288775**	A	**Gln110His**	Screening	99	81	12	0.27	-	-	-	-	95	83	14	0.29
	**rs146057680**	C	**Asp234Val**	Screening	192	0	0	0	-	-	-	-	191	1	0	0.003
	**rs773358289**	A	**Pro287Ser**	Screening	192	0	0	0	-	-	-	-	191	1	0	0.003
	**rs747005297**	C	**His292Arg**	Screening	191	1	0	0.003	-	-	-	-	192	0	0	0
	rs1145086	T	Leu418=	Screening	69	93	30	0.40	-	-	-	-	68	94	30	0.40

While the synonymous variant rs1136165 (p.Arg152=) was shown to have a sex-dimorphic effect on BMI (see Figures 2, 3; Supplementary Table S2), similar frequencies were found in our cases with severe obesity or AN as well as in healthy-lean controls (see Table 3). Hence, we were unable to detect associations of the T-allele (based on the forward strand) at rs1136165 with obesity (*p* = 0.72) or AN (*p* = 0.63) in our screening group (see Supplementary Table S4 and Supplementary Table S5). Nevertheless, *in silico* tools predicted a putative splice site-altering effect (see Table 4).

**TABLE 4:** *In silico* predictions of splice site alterations and pathogenicity of the detected variants in *CKB*, *CKMT1B,* and *GATM*. To analyze the putative effects of variants on splicing products, the tools ESEfinder (Cartegni et al., 2003) and Spliceman (Lim and Fairbrother, 2012) were applied. Predictions of pathogenicity and a conservation score were obtained by MutationTaster 2021 (Steinhaus et al., 2021). Non-synonymous variants are shown in bold.

Gene	Variant	Amino acid exchange	ESEfinder	Spliceman	MutationTaster2021	
			Splice site changes	Splice site—rank (%)	Prediction	PhyloP score
*CKB*	rs762206402	Pro112=	Changed	55	Neutral	−0.723
	rs1136165	Arg152=	Changed	70	Neutral	−0.515
	rs200890896	Ala175=	Changed	73	Neutral	0.247
	**rs146047573**	**Tyr269Cys**	Changed	66	Neutral	2.167
	rs1803283	Glu364=	Changed	60	Neutral	1.733
*CKMT1B*	g.43595061C/T	Thr79=	Changed	58	Neutral	1.104
	g.43595385C/T	Asp120=	Not changed	65	Neutral	−0.388
	g.43595451G/A	Thr142=	Changed	77	Neutral	−0.731
	**rs1230355611**	**Arg184Stop**	Changed	63	Deleterious	2.353
	**rs758572075**	**Val191Ala**	Changed	70	Neutral	1.217
	rs9571	Lys352=	Not changed	64	Neutral	0.774
	rs13234	Ala362=	Changed	71	Neutral	0.430
	rs149544188	Arg399=	Changed	66	Neutral	2.638
	rs144820945	Gly403=	Changed	57	Neutral	−0.249
*GATM*	**rs1288775**	**Gln110His**	Not changed	47	Neutral	0.902
	**rs146057680**	**Asp234Val**	Not changed	63	Deleterious	4.283
	**rs773358289**	**Pro287Ser**	Changed	68	Deleterious	4.949
	**rs747005297**	**His292Arg**	Changed	58	Deleterious	4.099
	rs1145086	Leu481=	Changed	64	Neutral	1.626

The rare, non-synonymous variant (rs146047573, p. Tyr269Cys) was detected heterozygously in a young girl from our screening group with severe obesity (BMI: 25.53 kg/m^2^; BMI-SDS: 2.23; 96th BMI percentile; age: 12.54 years) and neither in patients with AN nor in healthy-lean controls (see Table 3). It was predicted to be conserved and might alter splice sites, while the overall pathogenicity was indicated to be benign (see Tables 4 and 5). However, it putatively destabilizes the protein (see Table 5). Consequently, we genotyped rs146047573 in additional independent confirmation samples of 781 families with severe obesity (child or adolescent with severe obesity and both biological parents), 320 children and adolescents with severe obesity, and 253 healthy-lean controls (see Table 2). Thereby, we detected one additional female with severe obesity carrying the mutation heterozygously (BMI: 35.85 kg/m^2^; BMI-SDS: 2.96; 100th BMI percentile; age: 16.60 years) and no further carriers among the controls (see Table 3). No obesity association for the alternative allele (C) of rs146047573 was detected in our study groups (*p* = 1; see Supplementary Table S4). However, a nominal association of this variant’s alternative allele with obesity was evident upon comparison of an alternative non-Finnish European control group extracted from gnomAD with the screening group of 192 children or adolescents with severe obesity (*p* = 0.03) and our larger study group, including our screening and confirmation group (total of 1,293 children or adolescents with severe obesity; *p* = 0.02; see Table 2; Supplementary Table S4).

**TABLE 5:** *In silico* predictions of putative effects of non-synonymous variants on protein structure. The effect predictions were obtained with the CUPSAT tool (Parthiban et al., 2006) utilizing the protein structures of *CKB* (PBD ID: 3DRE; Bong et al., 2008), *CKMT1B* (PBD ID:1QK1; Eder et al., 2000) and *GATM* (PBD ID: 2JDW; Humm et al., 1997) and the PANTHER-PSEP tool (Tang and Thomas, 2016). As no full-length PBD protein structure was available for *CKMT1B*, we could not determine the impact of rs149544188 (^a^). As only non-synonymous variants could be predicted, the non-sense variant rs1230355611 (p. Arg184Stop) in *CKMT1B* was not analysed.

Gene	Variant	Amino acid exchange	Cupsat			Panther-PSEP		
			Protein stability	Protein torsion	ΔΔG (kcal/mol)	Preservation time	Prediction	Probability of a deleterious effect
*CKB*	rs146047573	Tyr269Cys	Destabilizing	Favorable	−2.93	361	Possibly damaging	0.5
*CKMT1B*	rs758572075	Val191Arg	Stabilizing	Favorable	0.96	324	Possibly damaging	0.5
	rs149544188^a^	Arg399His	*NA* ^*a*^	*NA* ^*a*^	*NA* ^*a*^	361	Possibly damaging	0.5
*GATM*	rs1288775	Gln110His	Destabilizing	Favorable	−0.3	6	Probably benign	0.02
	rs146057680	Asp234Val	Destabilizing	Favorable	−1.21	1,368	Probably damaging	0.86
	rs773358289	Pro287Ser	Destabilizing	Favorable	−0.81	797	Probably damaging	0.74
	rs747005297	His292Arg	Stabilizing	Unfavorable	0.06	3,806	Probably damaging	0.89

The rare synonymous variant rs200890896 (p.Ala175=) was exclusively detected in one heterozygous female with lifetime AN (BMI: 19.53 kg/m^2^; BMI-SDS: 1.84; age: 45.68 years see Table 3). It was predicted to be benign while still possessing the potential to alter splicing products (see Table 4). Within our study groups, no association with AN could be ascertained (*p* = 1; see Supplementary Table S5). However, a nominal AN association of the variant’s G allele was found (*p* = 0.02) when consulting an exclusively female control group extracted from gnomAD (see Supplementary Table S3 and Supplementary Table S5).

An additional rare synonymous variant (rs762206402; p.Pro112=) indicated to exert non-pathogenic effects (see Table 4) was detected in one heterozygous adolescent with severe obesity (BMI: 39.12 kg/m^2^; BMI-SDS: 3.08; 100th BMI percentile; age: 15.22 years see Table 3) and not in any other group. The infrequent C-allele of rs762206402 was nominally associated with obesity (*p* = 0.006) when analyzing the alternative gnomAD control group (see Supplementary Tables S3, S4). Additionally, one synonymous variant (rs1803283, p.Glu364 =) was frequently found (MAF ∼40%) in all three investigated screening groups (see Table 3). Non-pathogenic but putatively splice site-altering effects were determined by *in silico* tools (see Table 4). Previously, a BMI association of rs1803283 was found in females (*p* = 1.71*10^–12^) and both sexes combined in a GWAS (*p* = 1.48*10^–16^; Pulit et al., 2019).

### 3.3 Mutation screen of *CKMT1B*

Nine variants in the protein-coding sequence of *CKMT1B* were detected in the mutation screen of 192 children and adolescents with severe obesity and 192 lean control individuals (see Table 3). Notably, deviations from the HWE were detected for the novel variant (p.Thr79=) in our screening group with severe obesity and rs13234 (p.Ala362b =) in control individuals. Accordingly, we re-checked and repeated our sequencing in the corresponding samples. The results did not differ from the initial analyses. For instance, for the novel variant p.Thr79=, we detected one heterozygous and one homozygous carrier (see Table 3), leading to deviations from the HWE. Nevertheless, as we were able to exclude genotyping errors, it is feasible that the deviation occurred by chance. Therefore, we retained these variants in the subsequent analyses. All other detected variants fulfilled the HWE.

In total, we observed six synonymous (novel p.Thr79=, novel p.Asp120=, novel p.Thr142=, rs9571, rs13234, and rs144820945), one non-sense (rs1230355611) and two non-synonymous variants (rs758572075 and rs149544188) in the CDS of *CKMT1B*. The non-synonymous variant rs758572075 leading to a non-conservative amino acid exchange (p.Val191Ala) was found in one individual with obesity (BMI: 35.79 kg/m^2^; BMI-SDS: 2.96; 100th BMI percentile; age: 16.72 years) and two healthy-lean controls, while the other non-synonymous variant, rs149544188 (p.Arg399His) was identified twice among the children and adolescents with severe obesity and once in the lean control group of the screening samples (see Table 3). Both non-synonymous variants are likely benign but potentially alter splice sites (see Table 4). Furthermore, rs758572075 was predicted to have a stabilizing effect on CKMT1B as well as a favorable impact on its torsion (see Table 5). The non-sense variant rs1230355611 (p.Arg184Stop) was exclusively detected in one lean control individual analyzed in our screening study group (see Table 3). The *in silico* analyses indicated a deleterious effect of this non-sense mutation (see Table 4).

To evaluate a potential relevance for weight regulation of the protein-altering variants, we genotyped the non-sense (rs1230355611) and both non-synonymous (rs758572075 and rs149544188) variants in additional independent confirmation study groups. Here, within the 781 families with severe obesity, 320 independent children and adolescents with severe obesity, and 253 additional controls (see Table 2), we detected the non-synonymous variant rs758572075 (p.Val191Ala) in one additional boy with obesity (BMI: 32.85 kg/m^2^; BMI-SDS: 2.57; 100th BMI percentile, age: 14.36 years). The boy was heterozygous for this variant, which the mother who had obesity herself transmitted (BMI: 38.03 kg/m^2^; BMI-SDS: 1.95, age: 42.94 years), while the lean father (BMI: 22.63 kg/m^2^; BMI-SDS: 1.54, age: 48.28 years) did not harbor the variant. No association of the C-allele of this SNP with obesity was detected within our screening (*p* = 1) and confirmation study groups (*p* = 1; both groups combined: *p* = 0.27) (see Supplementary Table S4).

Moreover, the non-synonymous variant rs149544188 (p.Arg399His) was detected heterozygously in eleven additional patients with obesity and five healthy-lean controls (see Table 3). Of the eleven cases with obesity, nine were derived from the index cases of the 781 trios with severe obesity. In five cases, the mother transmitted the mutant allele, while the paternal allele was inherited in another four. In an additional 16 families, the child or adolescent with obesity was not a carrier of rs149544188, but one parent harbored the variant heterozygously (not shown). Six fathers and ten mothers were mutation carriers without transmitting the respective allele to their offspring. Consequently, the transmission rate for the mutant A-allele was 36%, indicating a transmission disequilibrium with a reduced transmission rate of the infrequent A-allele (p_chi-square_ = 0.16).

Furthermore, the non-sense variant rs1230355611 (p.Arg184Stop) was observed heterozygously in one girl with severe obesity (BMI: 31.34 kg/m^2^; BMI-SDS: 2.52; 99.fourth BMI percentile, age: 14.62 years; see Table 3). It was transmitted from the normal-weight mother (BMI: 24.98 kg/m^2^; BMI-SDS: 0.11; age: 45.12 years) to the offspring with severe obesity. There was no evidence of an obesity-predisposing effect of this variant (screening group: *p* = 1; confirmation group: *p* = 1; both groups: *p* = 0.45; see Supplementary Table S4).

Evidence for further phenotype-related associations was lacking (see Supplementary Table S4). Only when considering an additional control study group extracted from gnomAD (see Supplementary Table S3), the G-allele of the synonymous rs9571 (p.Lys352 =) was nominally associated (*p* = 0.00001) with obesity (see Supplementary Table S4). This variant was detected with the same frequency in our 192 patients with severe obesity and healthy-lean controls (MAF = 0.01; see Table 3).

### 3.4 Mutation screen of *GATM*

Sequencing of the coding region of *GATM* in 192 children and adolescents with severe obesity and 192 healthy-lean controls revealed one synonymous (rs1145086; p.Leu418=) and four non-synonymous variants (rs1288775, p.Gln110His; rs146057680, p.Asp234Val; rs773358289, p.Pro287Ser; and rs747005297, p.His292Arg; see Table 3). All identified variants fulfilled the HWE and were detected heterozygously. The missense variant rs1288775 and the synonymous SNP rs1145086 were frequently detected in children and adolescents with severe obesity and healthy-lean controls (MAF >20%; see Table 3). Additionally, two rare non-synonymous variants (rs146057680, p.Asp234Val, and rs773358289, p.Pro287Ser) were observed exclusively among controls. One non-synonymous variant, rs747005297 (p.His292Arg), was exclusively identified in a boy with severe obesity from our study group (BMI: 29.55 kg/m^2^; BMI-SDS: 2.90; 100th BMI percentile; age: 9.92 years). *In silico* analyses predicted the potential to affect splice sites and exhibit an overall deleterious effect (see Table 4). All non-synonymous SNPs were classified as either protein destabilizing or unfavorable regarding GATM’s torsion (see Table 5). Except for rs1288775 (p.Gln110His), all other non-synonymous variants were predicted to be deleterious (see Table 4). However, we were unable to find evidence for nominal associations of the variants localized in *GATM* with obesity (see Supplementary Table S4). But, when investigating the additional control cohort of Europeans extracted from gnomAD (see Supplementary Table S3), a nominal obesity association of the alternative C-allele of rs747005297 (*p* = 0.009) was observed (see Supplementary Table S4).

### 3.5 Correlations between genes and clinical parameters in visceral and subcutaneous adipose tissue

To complement our results with transcriptional data, we used RNA-seq data from 1,479 individuals of the LOBB. Based on these data, we analyzed correlations between *CKB*, *CKMT1B*, and *GATM* expression levels and specific metabolic and anthropometric parameters in both VAT and SAT of participants with and without obesity.

The unadjusted correlation analyses (*p* < 0.05) suggested relationships between several anthropometric and metabolic parameters and our genes of interest. In VAT, *CKB* correlated positively with *CKMT1B* (n = 1,479; *ρ* = 0.43; *p* < 0.001) and *GATM* (n = 1,479; *ρ* = 0.12; *p* < 0.001) as well as with body fat (n = 669; *ρ* = 0.11; *p* = 0.05), waist (n = 244; *ρ* = 0.16; *p* = 0.01), and non-esterified fatty acids (NEFA; n = 44; *ρ* = 0.30; *p* = 0.05; see Supplementary Figure S1). In the SAT, inverse correlations were detected. *CKB* was negatively correlated with *CKMT1B* (n = 1,479; *ρ* = −0.26; *p* < 0.001) and *GATM* (n = 1,479; *ρ* = −0.37; *p* < 0.001) as well as BMI (n = 1,479; *ρ* = −0.06; *p* = 0.01), homeostatic model assessment for insulin resistance (HOMA-IR; n = 416; *ρ* = −0.11; *p* = 0.02), NEFA (n = 44; *ρ* = −0.35; *p* = 0.02), and C-reactive protein (n = 1,435; *ρ* = −0.07; *p* = 0.005; see Supplementary Figure S1). *CKB* correlated negatively with hemoglobin A1C (hbA1C) in VAT (n = 790; *ρ* = −0.07; *p* = 0.04) and SAT (n = 790; *ρ* = −0.08; *p* = 0.03).

**FIGURE 4:**
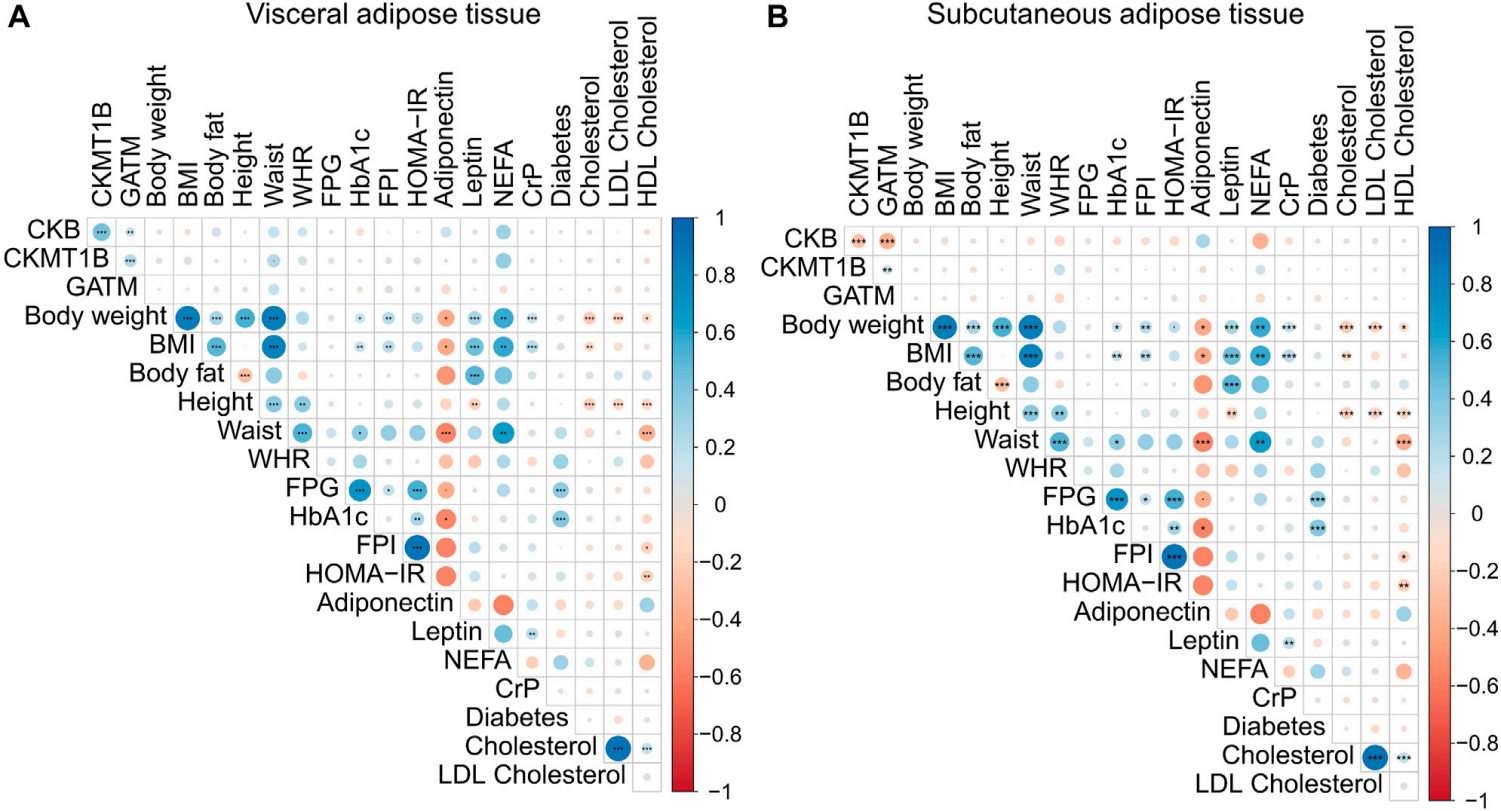
Correlations of *CKB*, *CKMT1B*, and *GATM* with clinical parameters. The presented data are based on RNA-sequencing data of omental visceral **(A)** and abdominal subcutaneous **(B)** adipose tissues from 1,479 individuals of the LOBB. The correlations were calculated with Pearson’s correlation coefficient. *p*-values were adjusted for multiple comparisons using Hommel’s method (Hommel, 1988). Positive correlations are shown in blue, while negative correlations are represented in red. The size of the dot refers to the degree of correlation. Significance; p_adj._ < 0.001 (***), p_adj._ < 0.01 (**), and p_adj._ < 0.05 (*). BMI: body mass index. CrP: c-reactive protein. FPI: fasting plasma insulin. FPG: fasting plasma glucose. HbA1C: hemoglobin A1C. HDL: high-density lipoprotein. HOMA-IR: homeostatic model assessment for insulin resistance. LDL: low-density lipoprotein. NEFA: non-esterified fatty acids. WHR: waist-to-hip ratio.

However, after correction for multiple inferences, only the inter-gene correlations were significant in both adipose tissues (see Figure 4 and Supplementary Figures S2, S3). As already seen in the data not corrected for multiple testing, all genes were positively correlated with *CKB* in VAT (*CKMT1B*: n = 1,479; *ρ* = 0.43; p_adj_ < 0.001; *GATM*: n = 1,479; *ρ* = 0.12; p_adj_ < 0.001; see Figure 4A and Supplementary Figure S3), while *CKB* was negatively correlated with *CKMT1B* (n = 1,479; *ρ* = - 0.26; p_adj._ < 0.001) and *GATM* (n = 1,479; *ρ* = −0.37; p_adj._ < 0.001) in SAT (see Figure 4B and Supplementary Figure S2). *CKMT1B* and *GATM* exhibited positive correlations in both VAT (n = 1,479; *ρ* = 0.21; p_adj._ < 0.001 see Figure 4 and Supplementary Figure S3) and SAT (n = 1,479; *ρ* = 0.12; p_adj._ < 0.001; see Figure 4 and Supplementary Figure S2).

Obesity and body weight regulation are influenced by sex hormones (e.g., Brettle et al., 2022; Vigil et al., 2022). As the mean age of our study groups was around 50 years (non-obese: 55.8 years old; participants with obesity: 46.9 years old) and most women experience their menopause by the age of 49 years (e.g., Davis et al., 2015), we subsequently excluded all participants above the age of 50 years (n = 465) to circumvent a putative menopause-based bias. Despite the removal of putatively post-menopausal women, differences in correlations were marginal (see Supplementary Figure S4). The inter-gene correlations in the visceral adipose tissue remained unchanged (see Supplementary Figure S4) from the analyses with all participants (see Figure 4A). Yet, the positive correlation between *CKMT1B* and *GATM* in the subcutaneous adipose tissue was not observed when participants above 50 years of age were excluded (see Supplementary Figure S4).

### 3.6 Gene expression of *CKB, CKMT1B* and *GATM* deviates between VAT and SAT

**FIGURE 5:**
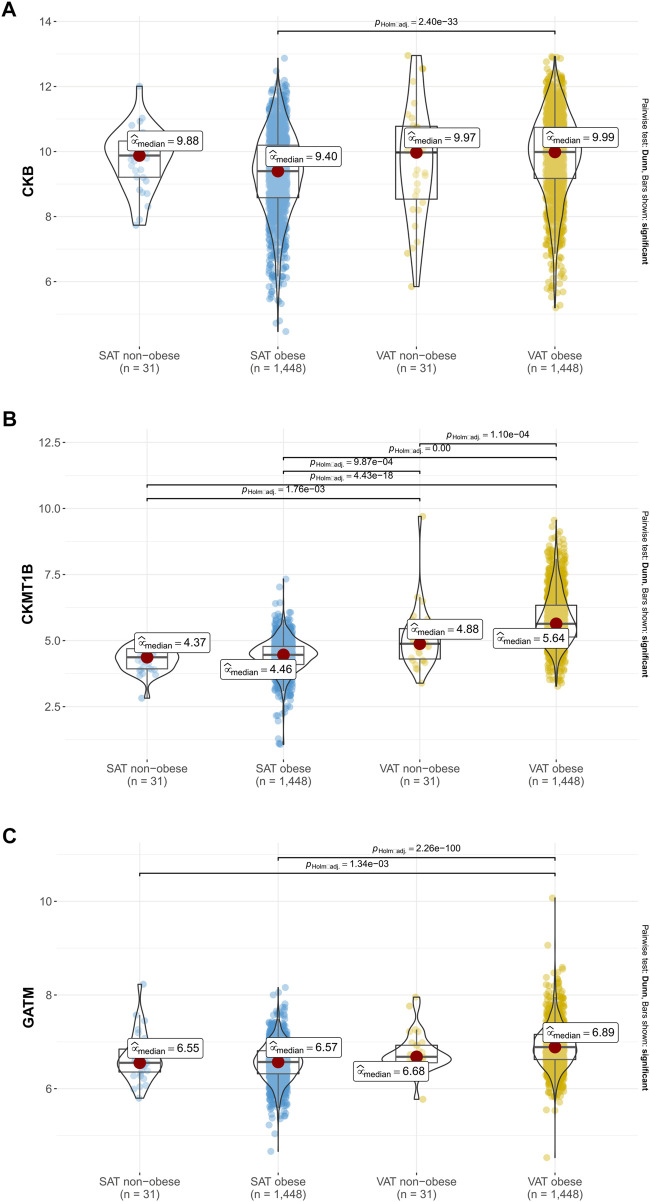
Gene expression of *CKB*, *CKMT1B* and *GATM* in probands with and without obesity. Expression data of *CKB* **(A)**, *CKMT1B* **(B)** and *GATM* **(C)** are based on 1,479 probands with and without obesity from the LOBB. As the data were not normally distributed in all instances, non-parametric Kruskal–Wallis one-way ANOVAs were performed to check whether the location parameters of the distributions were the same in each group. The corresponding effect size was calculated as epsilon square, while pairwise comparisons were performed with Dunn’s test. *p*-values were adjusted based on Hommel’s method (Hommel, 1988). SAT: subcutaneous adipose tissue. VAT: visceral adipose tissue.

Considering the inverse correlations of *CKB* with *CKMT1B* and *GATM* in visceral and subcutaneous adipose tissue (see Figure 4), we subsequently performed between-subject comparisons of non-obese participants and individuals with obesity from the LOBB for all three genes studied. Increased gene expression levels in the VAT of individuals with obesity in comparison to the SAT of this study group were found for *CKB* (p_adj._ < 0.001 see Figure 5A), *CKMT1B* (p_adj._ < 0.001; see Figure 5B), and *GATM* (p_adj._ < 0.001; see Figure 5C)*.* Deviations between participants with and without obesity in the VAT were exclusively observed for *CKMT1B* (p_adj._ < 0.001, see Figure 5B).

To again prevent bias due to the menopause, we excluded females with an age above 50 years. Again, solely minor differences were detected (see Supplementary Figure S5). Thus, the VAT of participants with and without obesity showed a higher expression of *CKB* than the SAT of patients with obesity (see Supplementary Figure S5).

## 4 Discussion

Previous studies have linked the creatine metabolism to BAT thermogenesis with the potential to counteract obesity (Streijger et al., 2005; Streijger et al., 2009; Kazak et al., 2015; Kazak et al., 2017; Pan et al., 2020; Rahbani et al., 2021). Transgenic mice depleted of CKB, CKMT1B, and GATM showed impaired creatine biosynthesis capacity affecting the murine body weight and resulting in a susceptibility to obesity (Streijger et al., 2005; Streijger et al., 2009; Kazak et al., 2017; Rahbani et al., 2021). Moreover, there is evidence of sex-specific effects regarding the regulation of body weight and BAT activity in rodents (Choi et al., 2011) as well as in humans (Hinney et al., 2017; Pulit et al., 2019). Therefore, we aimed to analyze the sex-specific effects of BMI-associated variants in the genes *CKB*, *CKMT1B* and *GATM*. Subsequently, we performed a mutation screen of these genes and *in silico* analyses. Furthermore, gene expression and correlation analyses based on data from the LOBB were performed.

### 4.1 Sex-stratified analyses

Considering these findings, we first explored the putative sex-stratified effects of SNPs located within *CKB*, *CKMT1B,* and *GATM* based on data from a BMI GWAS (Pulit et al., 2019). This analysis revealed one common SNP (rs1136165, p.Arg152 =) located in the genomic region of *CKB* with a sex-dimorphic effect. The BMI-increasing effect (beta-value derived from GWAS = 0.02; Pulit et al., 2019) was shown to be stronger in females than in males. In the 500 kb adjacent region of *CKB*, more than 4% of the GWAS SNPs revealed a BMI association. Given that enhancers may have a distance of more than 1 million base pairs to the gene’s promoter they affect (Nizovtseva et al., 2017), it is feasible to assume that some of these BMI-associated SNPs in the *CKB* adjacent region might disrupt enhancer sequences pertaining to the gene and eventually alter its transcription. In contrast, we did not detect evidence for SNPs exerting any effects, including sex-specific effects on *CKMT1B* and *GATM*. Moreover, by analyzing data from the latest AN GWAS (Watson et al., 2019), we could not determine variants associated with AN in the regions of interest (*CKB*, *CKMT1B*, *GATM,* and adjacent regions).

### 4.2 Mutation screen and confirmation studies

The CDSs of *CKB*, *CKMT1B,* and *GATM* were subsequently screened for variants in 192 children and adolescents with severe obesity as well as 192 healthy-lean controls. Furthermore, considering the abovementioned sex-specific GWAS hit, the observation that (1) AN and BMI share certain loci (Bulik-Sullivan et al., 2015; Hinney et al., 2017; Zheng et al., 2022) and 2) AN mainly affects females (Hübel et al., 2019; Mitchell and Peterson, 2020), we additionally screened the coding regions of *CKB* as the most likely target regarding creatine metabolism in 192 female patients with AN.

We identified five genetic variants in the coding regions of *CKB* and *GATM* each, while the CDS of *CKMT1B* revealed nine variants. The presence of infrequent non-synonymous variants in *CKB* and *CKMT1B* (rs146047573, rs758572075, rs1230355611, and rs149544188) was confirmed in independent study groups comprising 781 families with severe obesity, 320 children and adolescents with severe obesity, and 253 healthy lean controls (see Table 2). No evidence for associations of these variants with either obesity or AN was found (see Supplementary Tables S4, S5). Moreover, three novel variants within *CKMT1B* were detected. As these variants have not previously been described in any other population or regarding other phenotypes, it is not unlikely that, even though no association was detected, these variants might have a putative relevance for obesity. Furthermore, we detected that the variant rs149544188 located in *CKMT1B* was nominally less frequently transmitted by a heterozygous parent to the child with obesity than expected (transmission rate of 36%, p_Chi-square_ = 0.16).

In our study groups, we were unable to determine associations of the detected variants with obesity or AN. Presumably, the comparatively small sample size (see Table 2) as well as the low minor allele frequencies (see Table 3) of the detected variants may account for the lack of associations. Thus, we used larger datasets from gnomAD’s non-Finnish European population as an alternative control group (see Supplementary Tables S3–S5). With this, the infrequent alleles of the variants rs76220640 (p.Pro112 =) and rs146047573 (p.Tyr269Cys) located in *CKB*, the *CKMT1B* variant rs9571's allele (p.Lys352 =) as well as the allele of the *GATM*-located variant rs747005297 (p.His292Arg) were nominally associated with obesity. The infrequent allele at rs200890896 (p.Ala175 =) exhibited a nominal association with AN.

Regarding the interpretation of findings in comparison to the gnomAD control group, it is important to note that no phenotypic data were available regarding the data in this database. The gnomAD database mainly includes genotype information from case‒control studies regarding conditions with an onset in adulthood, such as cardiovascular diseases (Karczewski et al., 2020). Since these diseases are comorbidities of an increased BMI and obesity (Williams et al., 2015; Blüher, 2019), our statistical results using this control cohort might be biased by the recruitment of participants with an elevated BMI. However, as our study group consisted of children and adolescents with severe obesity at the extremes of the BMI distribution in the general population, we can assume that the gnomAD control group included only a few individuals with a similar degree of obesity. Finally, we cannot exclude confounding with respect to ethnic background upon the comparison of our German study groups with the gnomAD control group.

### 4.3 *In silico* analyses

Most of the identified non-synonymous variants in the genes of interest were predicted to impact the protein structure. Previously, alterations in the biochemical and biophysical properties of CKB caused by distinct genetic variations leading to amino acid substitutions have been analyzed (Li et al., 2012). Eight non-synonymous variants in *CKB* were studied. The results demonstrated that half of the variants’ amino acid substitutions affected the catalytic efficiency. For instance, the resultant amino acid exchange of rs13558 (p.Lys267GLu) increases the enzymatic activity by ∼ 30% compared to CKB containing the wild-type lysine (Li et al., 2012). Further amino acid substitution due to other SNP alleles conversely impaired the catalytic efficiency. In mice, some mutated alleles even implied reduced thermal stability below the physiological temperature of 37 °C (Li et al., 2012). Li et al. (2012) suspected that this impairment is related to thermoregulation in the double-KO mouse model (Streijger et al., 2005; Streijger et al., 2009; Li et al., 2012). Our *in silico* analyses predominantly pointed to benign effects, while the majority of variants exhibited the potential to alter splicing products. Only four non-synonymous variants were predicted to be deleterious overall (see Table 4). One of these variants (rs747005297) located in *GATM*, which was nominally associated with obesity (based on the alternative control group from gnomAD), was indicated to be protein stabilizing but unfavorable for GATM’s torsion (see Table 5). However, *in silico* analyses can merely indicate putative consequences of genetic variants and are no substitute for subsequent *in vitro* studies, which might reveal underlying pathological mechanisms. Particularly considering findings regarding the double-KO mouse model discussed above (Streijger et al., 2005), it is conceivable that polymorphisms in the genes studied here might have an influence on body weight in humans as well. Additionally, as mouse models already suggest compensatory processes between creatine kinases (Streijger et al., 2005), it is possible that mutations in either kinases or even all three genes would have a more severe impact on BMI.

### 4.4 Gene expression and correlation analyses

By analyzing RNA-seq data of VAT and SAT of 1,479 individuals (1,448 with obesity and 31 non-obese) of the Leipzig Obesity Biobank, we detected that all three genes of interest (*CKB*, *CKMT1B* and *GATM*) showed positive correlations with each other in visceral fat. However, a negative correlation of *CKB* with *CKMT1B* and *GATM* was observed in SAT. Further correlations with anthropometric or metabolic parameters, such as BMI or body fat, were not significant after correction for multiple comparisons. For *CKB*, *CKMT1B* and *GATM*, subsequent analyses revealed gene expression differences between VAT and SAT in 1,448 adipose tissue donors with obesity. We found consistently higher gene expression levels of the three genes in VAT than in SAT. Additionally, VAT *CKMT1B* expression was higher in patients with obesity in comparison to individuals with a BMI lower than 30 kg/m^2^.

As expected for genes belonging to the creatine metabolism pathway (Kazak et al., 2015; Kazak et al., 2017), we detected positive correlations between the genes in VAT. Yet, negative correlations among the three genes were found in SAT. Moreover, we found positive correlations between uncorrected gene expression of *CKB*, *CKMT1B*, and *GATM* with body fat in VAT and a negative correlation with BMI in SAT. After correcting for multiple testing, however, these correlations were not significant which indicate small effects that require even larger sample sizes.

*CKB* was negatively correlated with the other two genes in SAT, while positive correlations were observed in VAT. Differences between the two fat depots are known (e.g., Korsic et al., 2012; Spoto et al., 2014; Ronquillo et al., 2019), and *CKB* may reflect fat depot specific expression signatures. Expression differences of creatine metabolism pathway genes may therefore reflect functional differences between visceral and subcutaneous fat depots that have been previously reported (Gesta et al., 2006; Spoto et al., 2014; Mittal, 2019; Norreen-Thorsen et al., 2022). Furthermore, global transcriptomic differences in VAT and SAT of individuals with obesity and healthy controls revealed differential expression profiles in a recent study (Ronquillo et al., 2019).

Further, after the removal of 465 female participants older than 50 years, deviations in correlation and gene expression were minimal in comparison to the analysis including all individuals. However, we could depict differences between the inter-gene correlation of *CKMT1B* and *GATM* in the subcutaneous adipose tissue when excluding participants above the menopausal age. Based on our data, we can assume that *CKB*, *CKMT1B* and *GATM* are influenced only slightly by sex hormones. Yet, one proteome study in BAT of rats from 2011 revealed 48 proteins exhibiting a sex-specific regulation. Numerous of these proteins affect thermogenesis, such as the creatine kinase, and are more highly expressed in female than male rats (Choi et al., 2011). In order to gain unambiguous evidence whether sex hormones impact the expression of those genes, further and, above all, more targeted analyses are necessary. However, the results of our gene correlation analyses need to be interpreted with caution as the associations were of a small to moderate size (Schober et al., 2018). Nevertheless, we detected adipose tissue-specific expression and correlations of our genes of interest indicating that the alternative creatine-dependent mechanism regulating thermogenesis might be more active in VAT.

## 5 Conclusion

Taken together, one GWAS-based variant (rs1136165) located in *CKB* related to creatine metabolism exhibits a sex-stratified BMI effect. In the subsequent mutation screen of the coding regions of *CKB*, *CKTM1B,* and *GATM* and a larger confirmation group, five variants in the CDS of each *CKB* and *GATM* and nine variants in the coding region of *CKMT1B* were detected. No associations of the detected variants with either obesity or AN were found within our study groups. The TDT in 781 samples derived from trios with severe obesity (child or adolescent with severe obesity and both biological parents) suggested a nominal protective effect for obesity of the A-allele at rs149544188 located in *CKMT1B*. A nominally reduced transmission rate of 36% was determined. Analyses of a larger control cohort based on gnomAD revealed nominal associations of the infrequent alleles of the variants rs762206402 (p.Pro112=), rs146047573 (p.Tyr269Cys), rs9571 (p.Lys352=) and rs747005297 (p.His292Arg) with obesity. The alternative allele at rs200890896 (p.Ala175=) was nominally associated with AN. With our subsequent gene expression and correlation analyses, we were able to show adipose-tissue specific correlations patterns of *CKB* with *CKMT1B* and *GATM*. Furthermore, we detected higher expression levels of all genes in VAT than in SAT. *CKMT1B*’s expression was even higher in individuals with obesity than non-obese individuals. Hence, our results help to pin down potential targets for subsequent *in vitro* analyses that could reveal the true impact of the identified variants and explain underlying mechanisms.

## Data Availability

The datasets for this article are not publicly available due to concerns regarding participant/patient anonymity. Requests to access the datasets should be directed to the corresponding author.
