# Supplementary material for: Genetic variants in genes involved in creatine biosynthesis in patients with severe obesity or anorexia nervosa
Source: Front Genet. 2023 Apr 10;14:1128133. doi: 10.3389/fgene.2023.1128133 (PMC10123275; doi:10.3389/fgene.2023.1128133)
Supplement: Supplementary file 1 [file DataSheet1.pdf]

*Supplementary Material*

# Genetic Variants in Genes Involved in Creatine Biosynthesis in Patients with Severe Obesity or Anorexia Nervosa

**Luisa S. Rajcsanyi*, Anne Hoffmann, Adhideb Ghosh, Birgit Matrisch-Dinkler, Yiran Zheng, Triinu Peters, Wenfei Sun, Hua Dong, Falko Noé, Christian Wolfrum, Beate Herpertz-Dahlmann, Jochen Seitz, Martina de Zwaan, Wolfgang Herzog, Stefan Ehrlich, Stephan Zipfel, Katrin Giel, Karin Egberts, Roland Burghardt, Manuel Föcker, Linus T. Tsai, Timo D. Müller, Matthias Blüher, Johannes Hebebrand, Raphael Hirtz and Anke Hinney**

*** Correspondence:** Luisa S. Rajcsanyi: luisa.rajcsanyi@uk-essen.de

**Supplementary Table 1: List of primers and screening methods for all investigated genes (*CKB*, *CKMT1B* and *GATM*).**

| Gene | Fragment | Forward primer (5' to 3') | Reverse primer (5' to 3') | Fragment length (bp) | Method |
|---|---|---|---|---|---|
| ***CKB*** | E2/E3 | CTGAGTGGTACGCGGGAG | CTCAGCACGTAGTTGGGGTC | 658 | PCR |
| | E4/E5 | TGACGTCACTGTCCCCGT | GAGAGGGAAAGCGGAGGTAG | 1015 | PCR |
| | E6 | GGGTTCTCCTGCTGTGAGG | GGACTGATGCTGGGGCAC | 378 | PCR |
| | E7/E8 | CTGTAGGGGTTTCAGGCAGG | GCAGGCCAAAATAGTTTA | 697 | PCR |
| ***CKMT1B*** | E2-E7 | CATCCTCCCTCCCTGTTC | CTTCTTTGCACTCTCTGTCA | 2655 | PCR |
| | E2 | CATCCTCCCTCCCTGTTC | TGATTATAGCCCTTCTCTGTT | 325 | Nested PCR |
| | E3/E4 | CCAAGGTTTACCCTCTTTCTA | GCTGTATAATGACCATGAGTC | 739 | Nested PCR |
| | E5-E7 | CTTCAATTCCAGTGCTTTCC | TCCTGGGTTGGTTGGTAAA | 931 | Nested PCR |
| | E8 | TGTCATTCATTCACAGCCAAGTT | TCCTGGGCTCAAGTAATCTGC | 457 | PCR |
| | E9 | CAGGTTCAGGGCTCTTTCAG | GCCCTCGCAGACCTATCCT | 251 | PCR |
| | E10 | CTCAGACTGTAGGAAGCAGAT | CCACCATTAGAAGTAACACAGAC | 317 | PCR |
| ***GATM*** | E1 | CACTTCGTCAGAAGTCGCGT | CCGCAGGATCGAGTGAGT | 506 | PCR |
| | E2 | GTATGGTAGCTGGGGACACG | GCTGAAGGGAAAGCAGTCAG | 573 | PCR |
| | E3 | GGAGCAAAAGGCCTCAAAAT | TTTGGAGCTTTCCCCTTACA | 504 | PCR |

| | | | | | |
|---|---|---|---|---|---|
| | E4 | GCCTGGCCCTATATAGACCTT | CTCTTTGTGGATCATTTAGAAAAGT | 326 | PCR |
| | E5 | GCATGTTTTAGTCTAGAACTGAGTAGT | CATTTAGAACCATTAGGAACCATGG | 700 | PCR |
| | E6 | AGTCTCTGAAACCTGAGAAAAGT | TTGCAAACACAAGTCCCAGC | 510 | PCR |
| | E7 | CAAAAATGATAGCGGACATGG | GGGCTGAACAAGCTATCTGC | 481 | PCR |
| | E8 | GAACCACAGGACTCCTCCAA | CAGGCTTACGACCCCTGTTA | 344 | PCR |

**Supplementary Table 2: SNPs extracted from the GWAS for BMI or AN in *CKB*, *CKMT1B* and *GATM*, their relationship with the traits and sex-specific effects.** This table lists the number of SNPs extracted from the GWAS for BMI (Pulit et al., 2019) and AN (Watson et al., 2019) located in *CKB*, *CKMT1B* and *GATM* and contiguous regions (± 500 kb) as well as significant associations with the respective phenotype. Due to the close proximity of *CKMT1B* and *GATM*, these genes were analysed jointly by examining just one adjacent ± 500 kb region (*; see Table 1). SNPs associated with the investigated traits exhibiting a z score > |3| were classified as sex-dimorphic (Khramtsova et al., 2019). NA: not available.

| Gene | Region | BMI GWAS | | | | | | | AN GWAS | | | | |
|---|---|---|---|---|---|---|---|---|---|---|---|---|---|
| | | Total SNPs | Significant SNPs in combined analysis | Significant SNPs in females | Significant SNPs in males | Sex-dimorphic BMI-associated SNPs (z score > \|3\|) | | | Total SNPs | Significant SNPs | Sex-dimorphic AN-associated SNPs (z score > \|3\|) | | |
| | | | | | | n | Highest z-score | z-score P-value | | | n | Highest z-score | z-score P-value |
| ***CKB*** | Gene region | 24 | 3 | 2 | 0 | 1 | \|3.196\| | 0.0014 | 5 | 0 | 0 | *NA* | *NA* |
| | ± 500 kb region | 10,967 | 302 | 467 | 0 | 225 | \|4.265\| | 0.00002 | 3,221 | 0 | 0 | *NA* | *NA* |
| ***CKMT1B**** | Gene region | 35 | 0 | 0 | 0 | 0 | *NA* | *NA* | 8 | 0 | 0 | *NA* | *NA* |
| | ± 500 kb region | 22,211 | 1 | 0 | 0 | 0 | \|0.375\| | 0.71 | 4,728 | 0 | 0 | *NA* | *NA* |
| ***GATM**** | Gene region | 256 | 0 | 0 | 0 | 0 | *NA* | *NA* | 78 | 0 | 0 | *NA* | *NA* |
| | ± 500 kb region | 22,211 | 1 | 0 | 0 | 0 | \|0.375\| | 0.71 | 4,728 | 0 | 0 | *NA* | *NA* |

**Supplementary Table 3: Alternative control groups extracted from gnomAD.** The alternative control groups were extracted from gnomAD v2.1.1 (Karczewski et al., 2020). The control population for the screening group with severe obesity encompassed all non-Finnish, European individuals of either sex, while the control population for the female patients with anorexia nervosa solely consisted of non-Finnish, European females. The genotype-specific data were calculated on the basis of the allele counts, allele number and number of homozygotes stated in gnomAD for the corresponding SNP. For certain variants, no gnomAD data were available ([a]). Furthermore, if the variant was not detected in the respective screening group (patients with severe obesity or AN), no test was performed ([b]). The AN cohort was exclusively screened for *CKB* ([c]). No phenotypic data regarding the population were available. 11: homozygous wild-type. 12: heterozygous. 22: homozygous mutant allele. MAF: minor allele frequency. *NA*: not available.

| Gene | Variant | Amino acid exchange | gnomAD controls for obesity | | | | gnomAD controls for AN | | | |
|---|---|---|---|---|---|---|---|---|---|---|
| | | | 11 | 12 | 22 | MAF | 11 | 12 | 22 | MAF |
| ***CKB*** | rs762206402 | Pro112Pro | 29506 | 0 | 0 | 0 | *NA*[b] | *NA*[b] | *NA*[b] | *NA*[b] |
| | rs1136165 | Arg152Arg | 4762 | 15541 | 12624 | 0.38 | 2006 | 6617 | 5520 | 0.38 |
| | rs200890896 | Ala175Ala | *NA*[b] | *NA*[b] | *NA*[b] | *NA*[b] | 27053 | 2 | 0 | 0.00004 |
| | rs146047573 | Tyr269Cys | 64126 | 10 | 0 | 0.00008 | *NA*[b] | *NA*[b] | *NA*[b] | *NA*[b] |
| | rs1803283 | Glu364Glu | 8473 | 29704 | 25749 | 0.36 | 3640 | 12966 | 11432 | 0.36 |
| ***CKMT1B*** | g.43595061C/T[a] | Thr79Thr | *NA*[a] | *NA*[a] | *NA*[a] | *NA*[a] | *NA*[c] | *NA*[c] | *NA*[c] | *NA*[c] |
| | g.43595385C/T[a] | Asp120Asp | *NA*[a] | *NA*[a] | *NA*[a] | *NA*[a] | *NA*[c] | *NA*[c] | *NA*[c] | *NA*[c] |
| | g.43595451G/A[a] | Thr142Thr | *NA*[a] | *NA*[a] | *NA*[a] | *NA*[a] | *NA*[c] | *NA*[c] | *NA*[c] | *NA*[c] |
| | rs1230355611 | Arg184Stop | 8644 | 0 | 0 | 0 | *NA*[c] | *NA*[c] | *NA*[c] | *NA*[c] |
| | rs758572075 | Val191Ala | 9066 | 31 | 1 | 0.002 | *NA*[c] | *NA*[c] | *NA*[c] | *NA*[c] |
| | rs9571 | Lys352Lys | 64235 | 41 | 0 | 0.0003 | *NA*[c] | *NA*[c] | *NA*[c] | *NA*[c] |
| | rs13234 | Ala362Ala | 63532 | 699 | 6 | 0.006 | *NA*[c] | *NA*[c] | *NA*[c] | *NA*[c] |
| | rs149544188 | Arg399His | 63685 | 840 | 14 | 0.007 | *NA*[c] | *NA*[c] | *NA*[c] | *NA*[c] |
| | rs144820945 | Gly403Gly | 63874 | 654 | 5 | 0.005 | *NA*[c] | *NA*[c] | *NA*[c] | *NA*[c] |
| ***GATM*** | rs1288775 | Gln110His | 34476 | 25365 | 4630 | 0.27 | *NA*[c] | *NA*[c] | *NA*[c] | *NA*[c] |
| | rs146057680 | Asp234Val | *NA*[b] | *NA*[b] | *NA*[b] | *NA*[b] | *NA*[c] | *NA*[c] | *NA*[c] | *NA*[c] |
| | rs773358289 | Pro287Ser | *NA*[b] | *NA*[b] | *NA*[b] | *NA*[b] | *NA*[c] | *NA*[c] | *NA*[c] | *NA*[c] |
| | rs747005297 [a] | His292Arg | 64530 | 2 | 0 | 0.00002 | *NA*[c] | *NA*[c] | *NA*[c] | *NA*[c] |
| | rs1145086 | Leu418Leu | 24189 | 30475 | 9541 | 0.39 | *NA*[c] | *NA*[c] | *NA*[c] | *NA*[c] |

**Supplementary Table 4: Statistical association testing of the detected variants and obesity.** The two-sided chi-square test or Fisher's exact test was performed with IBM SPSS Statistics (version 28.0.0.0) on the basis of the genotype counts (see Table 3 and Supplementary Table 3). Here, the results of the association testing for the screening and confirmation groups with severe obesity as well as the healthy-lean controls and the alternative gnomAD control cohort (see Tables 2 and Supplementary Table 3) are represented. The minor alleles are given with respect to the forward strand ([a]). If the variant was not detected in the respective study group, no test was performed ([b]; see Table 3). Furthermore, certain variants were not included in gnomAD ([c]; see Supplementary Table 3); thus, no statistical testing could be performed. Nominal associations are shown in bold ($p < 0.05$). The GWAS-derived p-values stated are based on the BMI-GWAS analyzing both sexes combined (Pulit et al., 2019). Again, several variants were not analyzed within this GWAS ([e]). GWAS: genome-wide association study. *NA*: no statistical test performed. p: p-value.

| Gene | Variant | Minor allele[a] | Study group | Obese vs. healthy-lean controls | | Obese vs. gnomAD controls | | BMI GWAS | |
|---|---|---|---|---|---|---|---|---|---|
| | | | | | | | | Pulit et al., 2019 | |
| | | | | Test | p | Test | p | Tested allele | p |
| ***CKB*** | **rs762206402** | C | **Screening** | Fisher's exact | 1 | Fisher's exact | **0.006** | *NA*[e] | *NA*[e] |
| | rs1136165 | G | Screening | Chi-square | 0.72 | Chi-square | 0.80 | T | $3.9*10^{-15}$ |
| | rs200890896 | G | Screening | *NA*[b] | *NA*[b] | *NA*[b] | *NA*[b] | *NA*[e] | *NA*[e] |
| | **rs146047573** | C | **Screening** | Fisher's exact | 1 | Fisher's exact | **0.03** | *NA*[e] | *NA*[e] |
| | | | Confirmation | Fisher's exact | 1 | Fisher's exact | 0.17 | | |
| | | | **Both** | Fisher's exact | 1 | Fisher's exact | **0.02** | | |
| | rs1803283 | C | Screening | Chi-square | 0.72 | Chi-square | 0.57 | T | $1.5*10^{-16}$ |
| ***CKMT1B*** | g.43595061C/T | T | Screening | Fisher's exact | 1 | *NA*[c] | *NA*[c] | *NA*[e] | *NA*[e] |
| | g.43595385C/T | T | Screening | Fisher's exact | 1 | *NA*[c] | *NA*[c] | *NA*[e] | *NA*[e] |
| | g.43595451G/A | A | Screening | Fisher's exact | 1 | *NA*[c] | *NA*[c] | *NA*[e] | *NA*[e] |
| | rs1230355611 | T | Screening | Fisher's exact | 1 | *NA*[b] | *NA*[b] | *NA*[e] | *NA*[e] |
| | | | Confirmation | Fisher's exact | 1 | Fisher's exact | 0.11 | | |
| | | | Both | Fisher's exact | 0.45 | Fisher's exact | 0.13 | | |
| | rs758572075 | C | Screening | Fisher's exact | 1 | Chi-square | 0.91 | *NA*[e] | *NA*[e] |
| | | | Confirmation | Fisher's exact | 1 | Chi-square | 0.35 | | |
| | | | Both | Fisher's exact | 0.27 | Chi-square | 0.50 | | |
| | **rs9571** | G | **Screening** | Fisher's exact | 1 | Fisher's exact | **0.00001** | *NA*[e] | *NA*[e] |
| | rs13234 | C | Screening | Chi-square | 0.42 | Chi-square | 0.13 | *NA*[e] | *NA*[e] |

| | | | | | | | | | |
|---|---|---|---|---|---|---|---|---|---|
| | rs149544188 | A | Screening | Fisher's exact | 1 | Chi-square | 0.93 | A | 0.21 |
| | | | Confirmation | Fisher's exact | 0.73 | Chi-square | 0.60 | | |
| | | | Both | Fisher's exact | 1 | Chi-square | 0.56 | | |
| | rs144820945 | G | Screening | Fisher's exact | 1 | Chi-square | 0.33 | G | 0.28 |
| ***GATM*** | rs1288775 | A | Screening | Chi-square | 0.88 | Chi-square | 0.75 | A | 0.007 |
| | rs146057680 | C | Screening | Fisher's exact | 1 | *NA*[b] | *NA*[b] | T | 0.41 |
| | rs773358289 | A | Screening | Fisher's exact | 1 | *NA*[b] | *NA*[b] | *NA*[e] | *NA*[e] |
| | **rs747005297** | C | **Screening** | Fisher's exact | 1 | Fisher's exact | **0.009** | *NA*[e] | *NA*[e] |
| | rs1145086 | T | Screening | Chi-square | 0.99 | Chi-square | 0.88 | A | 0.001 |

**Supplementary Table 5: Statistical association testing of *CKB*-located variants and anorexia nervosa.** Here, the chi-square test or Fisher's exact test was performed using IBM SPSS Statistics (version 28.0.0.0) on the basis of the genotype counts (see Table 3 and Supplementary Table 3). Here, only the results of the association testing for anorexia nervosa in all study groups investigated for this phenotype (see Table 3 and Supplementary Table 3) are represented. Nominally significant p-values are shown in bold ($p < 0.05$). The minor alleles are given with respect to the forward strand ([a]). If the variant was not detected in the AN study group, no test was performed ([b]). The presented p-value was extracted from the latest GWAS for AN (Watson et al., 2019). Again, several variants were not analyzed in this GWAS ([c]). p: p-value. *NA*: no statistical test performed.

| Variant | Alternative allele | AN vs. healthy-lean | | AN vs. gnomAD controls | | AN GWAS | |
|---|---|---|---|---|---|---|---|
| | | | | | | Watson et al., 2019 | |
| | | Test | p | Test | p | Tested allele | p |
| rs762206402 | C | *NA*[b] | *NA*[b] | *NA*[b] | *NA*[b] | *NA*[c] | *NA*[c] |
| rs1136165 | C | Chi-square | 0.63 | Chi-square | 0.97 | G | 0.07 |
| **rs200890896** | G | Fisher's exact | 1 | Fisher's exact | **0.02** | *NA*[c] | *NA*[c] |
| rs146047573 | C | *NA*[b] | *NA*[b] | *NA*[b] | *NA*[b] | *NA*[c] | *NA*[c] |
| rs1803283 | C | Chi-square | 0.87 | Chi-square | 0.94 | C | 0.34 |

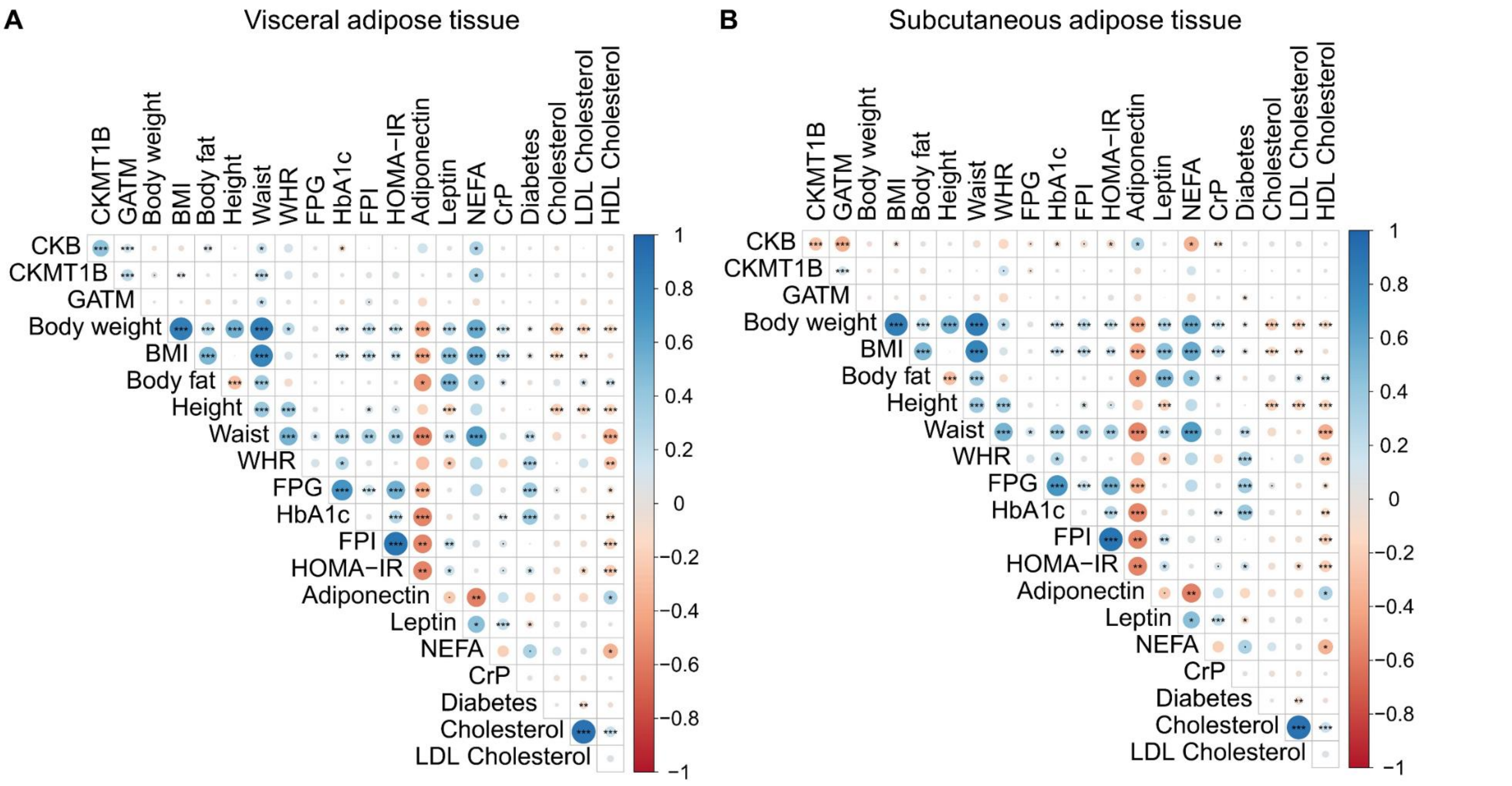


**Supplementary Figure 1: Unadjusted correlations of *CKB*, *CKMT1B*, and *GATM* in visceral and subcutaneous fat.** The presented data are based on RNA-sequencing data of 1,479 subjects of the Leipzig Obesity Biobank. For each individual, data for the visceral (A) and subcutaneous (B) adipose were available. The correlations were calculated with Pearson's correlation coefficient. P-values are not adjusted for multiple comparisons. Positive correlations are shown in blue, while negative correlations are represented in red. The size of the dot refers to the degree of correlation. $p < 0.001$ (***), $p < 0.01$ (**), and $p < 0.05$ (*). BMI: body mass index. CrP: c-reactive protein. FPI: fasting plasma insulin. FPG: fasting plasma glucose. HbA1C: hemoglobin A1C. HDL: high-density lipoprotein. HOMA-IR: homeostatic model assessment for insulin resistance. LDL: low-density lipoprotein. NEFA: nonesterified fatty acids. WHR: waist-to-hip ratio.

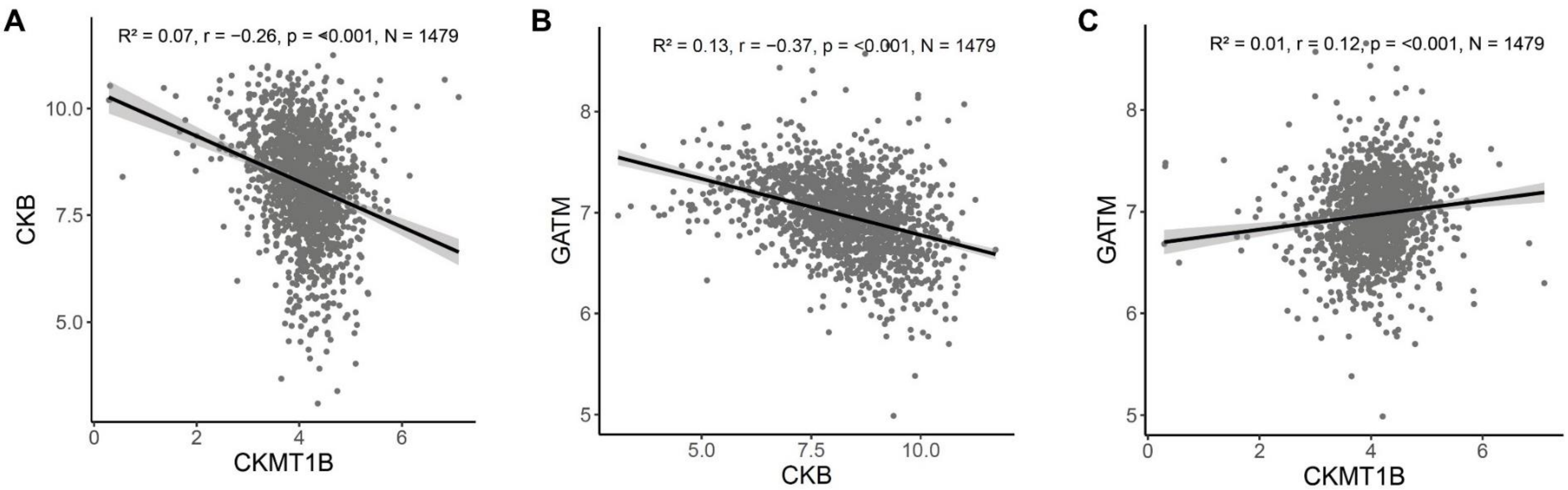


**Supplementary Figure 2: Multiple testing adjusted gene correlations in the subcutaneous adipose tissue.** Correlations between the three genes of interest are shown. These correlations were calculated with Pearson's correlation coefficient. P-values are adjusted for multiple inference.

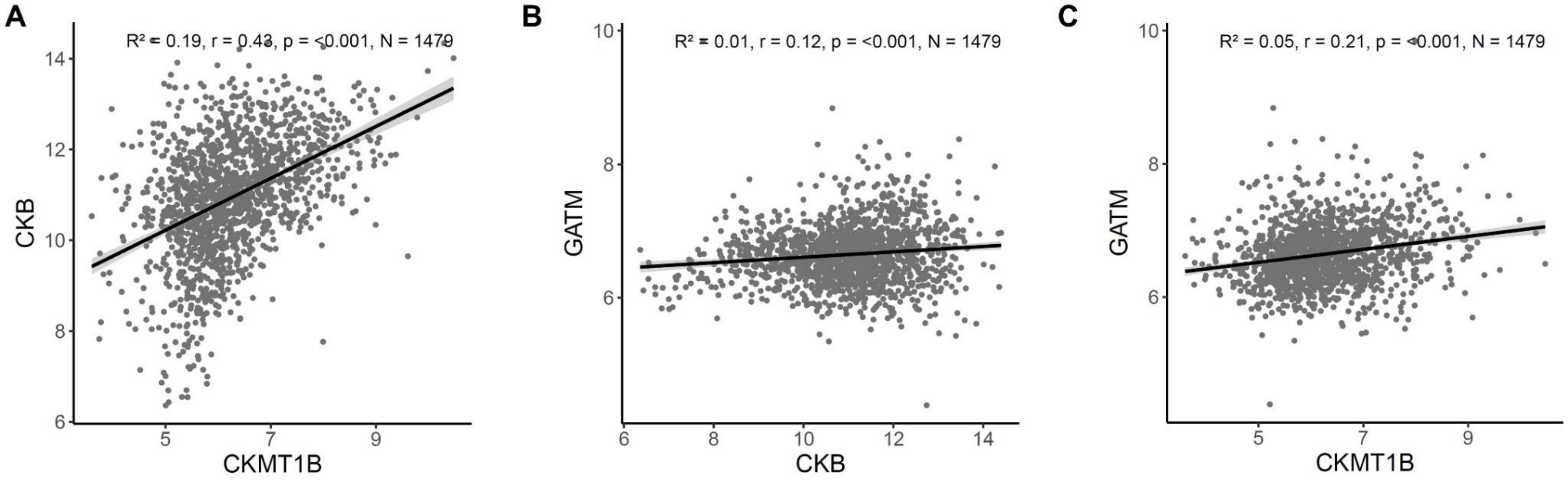


**Supplementary Figure 3: Multiple testing adjusted gene correlations in visceral adipose tissue.** Correlations between the three genes of interest are shown. These correlations were calculated with Pearson's correlation coefficient. P-values are adjusted for multiple inference.

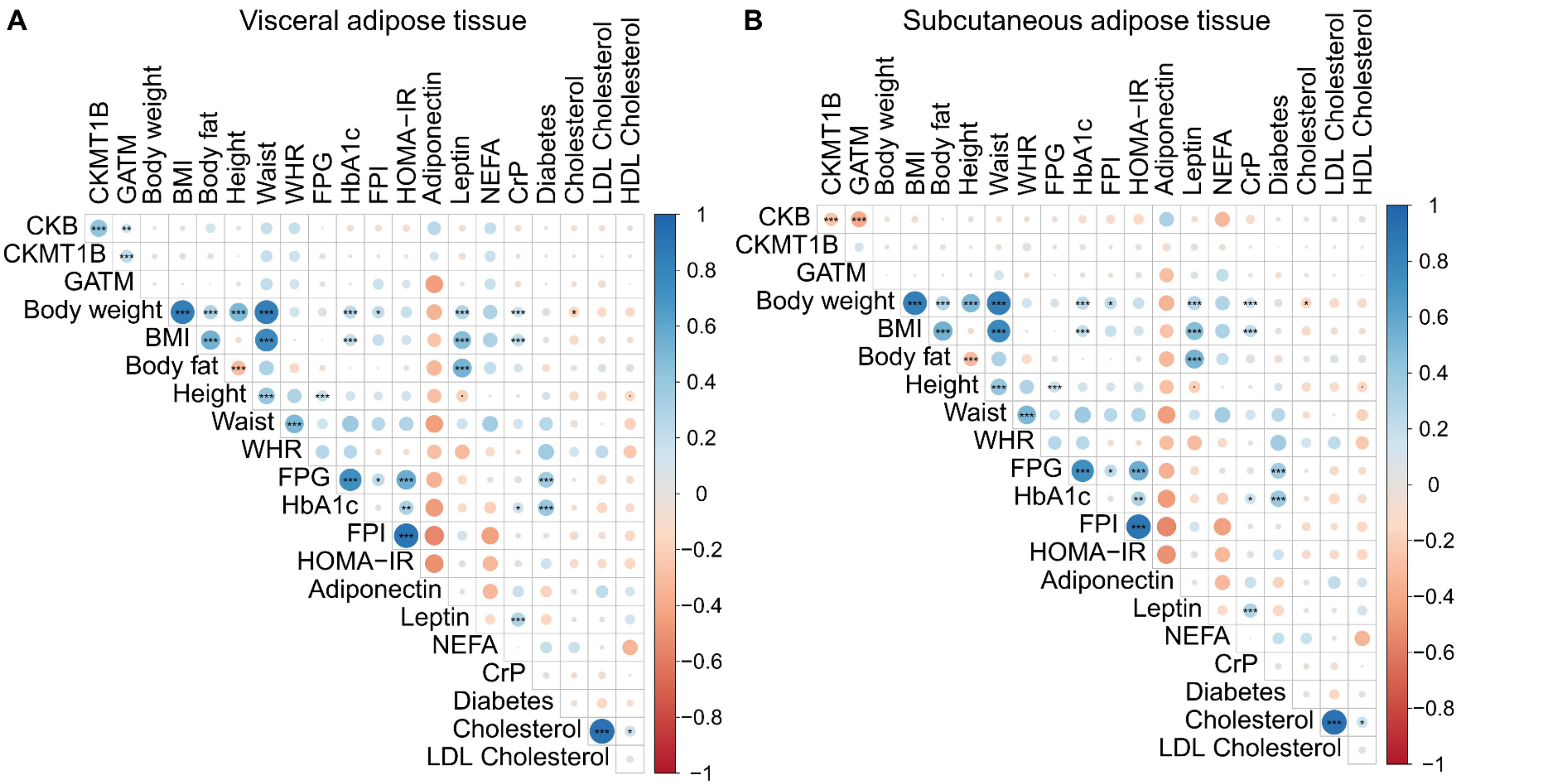


**Supplementary Figure 4: Adjusted correlations of *CKB*, *CKMT1B* and *GATM* in VAT and SAT in participants under 50 years of age.** The present data are based on RNA-sequencing data of the Leipzig Obesity Biobank. Here, exclusively participants under the age of 50 were investigated to prevent a post-menopausal bias (n = 1,014). For each included participant, data for the visceral (A) and subcutaneous (B) adipose tissue was analysed. Correlations were calculated with Pearson's correlation coefficient and p-values were adjusted for multiple comparisons using the Hommel's method (Hommel, 1988). Positive correlations are shown in blue. Negative correlations are represented in red. The size of the dots refer to the degree of correlation. p <0.001 (***), p <0.01 (**), and p <0.05 (*). BMI: body mass index. CrP: c-reactive

protein. FPI: fasting plasma insulin. FPG: fasting plasma glucose. HbA1C: hemoglobin A1C. HDL: high-density lipoprotein. HOMA-IR: homeostatic model assessment for insulin resistance. LDL: low-density lipoprotein. NEFA: nonesterified fatty acids. WHR: waist-to-hip ratio.

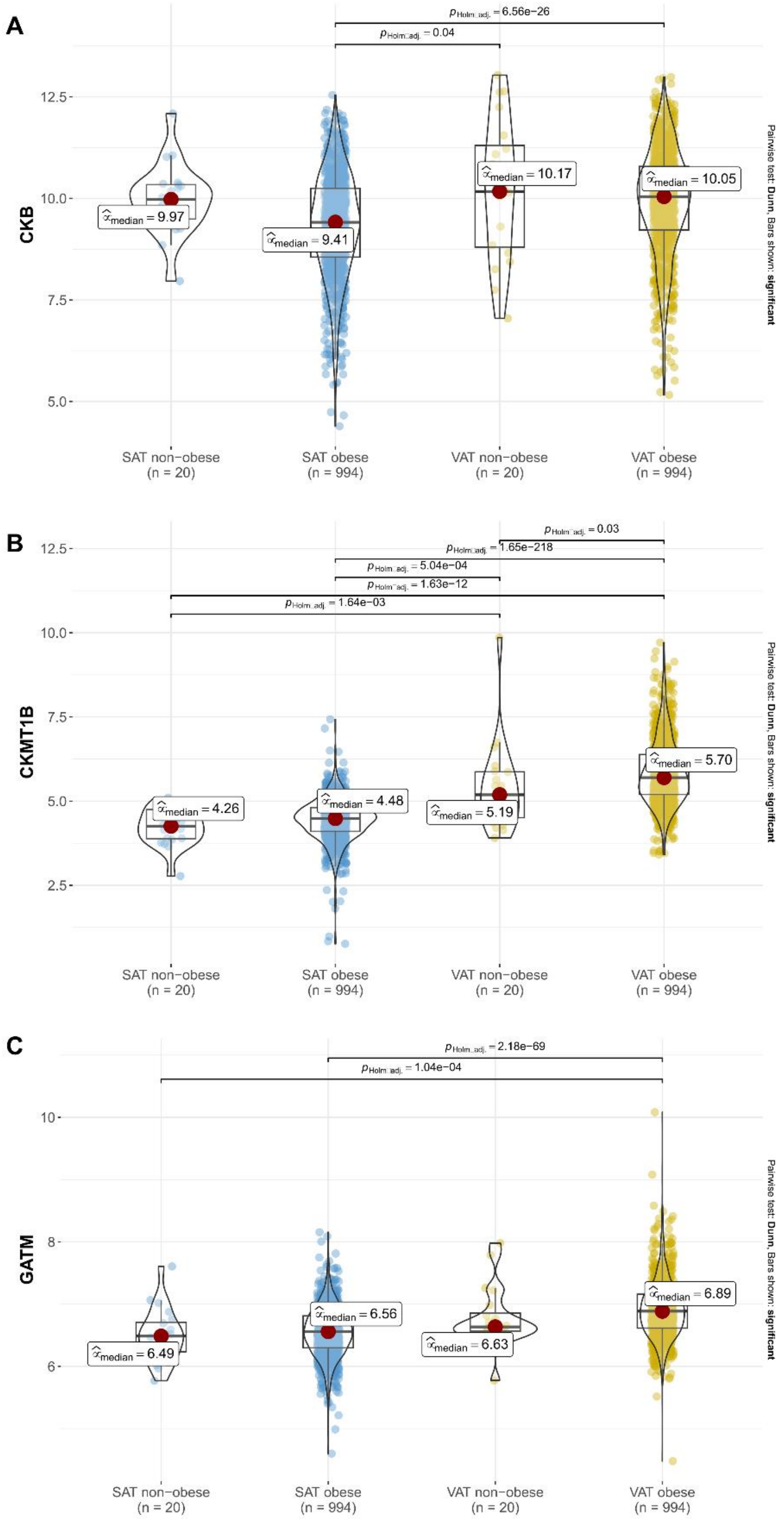


**Supplementary Figure 5: Gene expression of *CKB, CKMT1B* and *GATM* in probands with and without obesity under the age of 50.** Here, the gene expression of *CKB* (A), *CKMT1B* (B) and *GATM* (C) are shown. The underlying data are based on probands with and without obesity under the age of

50 years included in the Leipzig Obesity Biobank (n = 1,014). Since the data was not always normally distributed, the non-parametric Kruskal-Wallis one-way ANOVA was performed. The respective effect size was calculated as epsilon square, while pairwise comparisons were performed with Dunn's test. P-values were adjusted based on Hommel's method (Hommel, 1988). SAT: subcutaneous adipose tissue. VAT: visceral adipose tissue.


HOMMEL, G. 1988. A stagewise rejective multiple test procedure based on a modified Bonferroni test. *Biometrika,* 75**,** 383-386.

KARCZEWSKI, K. J., FRANCIOLI, L. C., TIAO, G., CUMMINGS, B. B., ALFOLDI, J., WANG, Q., COLLINS, R. L., LARICCHIA, K. M., GANNA, A., BIRNBAUM, D. P., GAUTHIER, L. D., BRAND, H., SOLOMONSON, M., WATTS, N. A., RHODES, D., SINGER-BERK, M., ENGLAND, E. M., SEABY, E. G., KOSMICKI, J. A., WALTERS, R. K., TASHMAN, K., FARJOUN, Y., BANKS, E., POTERBA, T., WANG, A., SEED, C., WHIFFIN, N., CHONG, J. X., SAMOCHA, K. E., PIERCE-HOFFMAN, E., ZAPPALA, Z., O'DONNELL-LURIA, A. H., MINIKEL, E. V., WEISBURD, B., LEK, M., WARE, J. S., VITTAL, C., ARMEAN, I. M., BERGELSON, L., CIBULSKIS, K., CONNOLLY, K. M., COVARRUBIAS, M., DONNELLY, S., FERRIERA, S., GABRIEL, S., GENTRY, J., GUPTA, N., JEANDET, T., KAPLAN, D., LLANWARNE, C., MUNSHI, R., NOVOD, S., PETRILLO, N., ROAZEN, D., RUANO-RUBIO, V., SALTZMAN, A., SCHLEICHER, M., SOTO, J., TIBBETTS, K., TOLONEN, C., WADE, G., TALKOWSKI, M. E., GENOME AGGREGATION DATABASE, C., NEALE, B. M., DALY, M. J. & MACARTHUR, D. G. 2020. The mutational constraint spectrum quantified from variation in 141,456 humans. *Nature,* 581**,** 434-443.

KHRAMTSOVA, E. A., HELDMAN, R., DERKS, E. M., YU, D., TOURETTE SYNDROME/OBSESSIVE-COMPULSIVE DISORDER WORKING GROUP OF THE PSYCHIATRIC GENOMICS, C., DAVIS, L. K. & STRANGER, B. E. 2019. Sex differences in the genetic architecture of obsessive-compulsive disorder. *Am J Med Genet B Neuropsychiatr Genet,* 180**,** 351-364.

PULIT, S. L., STONEMAN, C., MORRIS, A. P., WOOD, A. R., GLASTONBURY, C. A., TYRRELL, J., YENGO, L., FERREIRA, T., MAROULI, E., JI, Y., YANG, J., JONES, S., BEAUMONT, R., CROTEAU-CHONKA, D. C., WINKLER, T. W., CONSORTIUM, G., HATTERSLEY, A. T., LOOS, R. J. F., HIRSCHHORN, J. N., VISSCHER, P. M., FRAYLING, T. M., YAGHOOTKAR, H. & LINDGREN, C. M. 2019. Meta-analysis of genome-wide association studies for body fat distribution in 694 649 individuals of European ancestry. *Hum Mol Genet,* 28**,** 166-174.

WATSON, H. J., YILMAZ, Z., THORNTON, L. M., HUBEL, C., COLEMAN, J. R. I., GASPAR, H. A., BRYOIS, J., HINNEY, A., LEPPA, V. M., MATTHEISEN, M., MEDLAND, S. E., RIPKE, S., YAO, S., GIUSTI-RODRIGUEZ, P., ANOREXIA NERVOSA GENETICS, I., HANSCOMBE, K. B., PURVES, K. L., EATING DISORDERS WORKING GROUP OF THE PSYCHIATRIC GENOMICS, C., ADAN, R. A. H., ALFREDSSON, L., ANDO, T., ANDREASSEN, O. A., BAKER, J. H., BERRETTINI, W. H., BOEHM, I., BONI, C., PERICA, V. B., BUEHREN, K., BURGHARDT, R., CASSINA, M., CICHON, S., CLEMENTI, M., CONE, R. D., COURTET, P., CROW, S., CROWLEY, J. J., DANNER, U. N., DAVIS, O. S. P., DE ZWAAN, M., DEDOUSSIS, G., DEGORTES, D., DESOCIO, J. E., DICK, D. M., DIKEOS, D., DINA, C., DMITRZAK-WEGLARZ, M., DOCAMPO, E., DUNCAN, L. E., EGBERTS, K., EHRLICH, S., ESCARAMIS, G., ESKO, T., ESTIVILL, X., FARMER, A., FAVARO, A., FERNANDEZ-ARANDA, F., FICHTER, M. M., FISCHER, K.,

FOCKER, M., FORETOVA, L., FORSTNER, A. J., FORZAN, M., FRANKLIN, C. S., GALLINGER, S., GIEGLING, I., GIURANNA, J., GONIDAKIS, F., GORWOOD, P., MAYORA, M. G., GUILLAUME, S., GUO, Y., HAKONARSON, H., HATZIKOTOULAS, K., HAUSER, J., HEBEBRAND, J., HELDER, S. G., HERMS, S., HERPERTZ-DAHLMANN, B., HERZOG, W., HUCKINS, L. M., HUDSON, J. I., IMGART, H., INOKO, H., JANOUT, V., JIMENEZ-MURCIA, S., JULIA, A., KALSI, G., KAMINSKA, D., KAPRIO, J., KARHUNEN, L., KARWAUTZ, A., KAS, M. J. H., KENNEDY, J. L., KESKI-RAHKONEN, A., KIEZEBRINK, K., KIM, Y. R., KLARESKOG, L., KLUMP, K. L., KNUDSEN, G. P. S., LA VIA, M. C., et al. 2019. Genome-wide association study identifies eight risk loci and implicates metabo-psychiatric origins for anorexia nervosa. *Nat Genet*, 51**,** 1207-1214.
